# Functional genomic analysis of constitutive and inducible defense responses to *Fusarium verticillioides* infection in maize genotypes with contrasting ear rot resistance

**DOI:** 10.1186/1471-2164-15-710

**Published:** 2014-08-25

**Authors:** Alessandra Lanubile, Alberto Ferrarini, Valentina Maschietto, Massimo Delledonne, Adriano Marocco, Diana Bellin

**Affiliations:** Istituto di Agronomia, Genetica e Coltivazioni erbacee, Università Cattolica del Sacro Cuore, Via Emilia Parmense 84, 29122 Piacenza, Italy; Dipartimento di Biotecnologie, Università degli Studi di Verona, Strada Le Grazie 15, 37134 Verona, Italy

**Keywords:** RNA-Seq analysis, *Fusarium* ear rot, Constitutive defense, Secondary metabolism, Candidate genes, *Zea mays*

## Abstract

**Background:**

*Fusarium verticillioides* causes ear rot in maize (*Zea mays* L.) and accumulation of mycotoxins, that affect human and animal health. Currently, chemical and agronomic measures to control *Fusarium* ear rot are not very effective and selection of more resistant genotypes is a desirable strategy to reduce contaminations. A deeper knowledge of molecular events and genetic basis underlying *Fusarium* ear rot is necessary to speed up progress in breeding for resistance.

**Results:**

A next-generation RNA-sequencing approach was used for the first time to study transcriptional changes associated with *F. verticillioides* inoculation in resistant CO441 and susceptible CO354 maize genotypes at 72 hours post inoculation. More than 100 million sequence reads were generated for inoculated and uninoculated control plants and analyzed to measure gene expression levels. Comparison of expression levels between inoculated vs. uninoculated and resistant vs. susceptible transcriptomes revealed a total number of 6,951 differentially expressed genes. Differences in basal gene expression were observed in the uninoculated samples. CO441 genotype showed a higher level of expression of genes distributed over all functional classes, in particular those related to secondary metabolism category. After *F. verticillioides* inoculation, a similar response was observed in both genotypes, although the magnitude of induction was much greater in the resistant genotype. This response included higher activation of genes involved in pathogen perception, signaling and defense, including WRKY transcription factors and jasmonate/ethylene mediated defense responses. Interestingly, strong differences in expression between the two genotypes were observed in secondary metabolism category: pathways related to shikimate, lignin, flavonoid and terpenoid biosynthesis were strongly represented and induced in the CO441 genotype, indicating that selection to enhance these traits is an additional strategy for improving resistance against *F. verticillioides* infection.

**Conclusions:**

The work demonstrates that the global transcriptional analysis provided an exhaustive view of genes involved in pathogen recognition and signaling, and controlling activities of different TFs, phytohormones and secondary metabolites, that contribute to host resistance against *F. verticillioides*. This work provides an important source of markers for development of disease resistance maize genotypes and may have relevance to study other pathosystems involving mycotoxin-producing fungi.

**Electronic supplementary material:**

The online version of this article (doi:10.1186/1471-2164-15-710) contains supplementary material, which is available to authorized users.

## Background

*Fusarium verticillioides* (Sacc.) Nirenberg (synonym *F. moniliforme* Sheldon, teleomorph *Gibberella moniliformis* Wineland) is a fungal pathogen that causes severe stalk rot and ear rot of maize and it is found in plant residues in almost every maize field at harvest [1]. In Southern Europe, *F. verticillioides* is the prevalent species in maize fields and it is the main causal agent of *Fusarium* ear rot [2, 3]. *F. verticillioides* is associated with maize in most stages of this plant’s growth cycle. The fungus is both a parasite and a saprophyte. Under ordinary plant growth condition, the hemibiotrophic *F. verticillioides* grows within the maize plant as an endophyte. Symptoms vary greatly depending upon plant genotype, environment and disease severity [4]. Interest in *F. verticillioides* is intense because the fungus can produce fumonisins as secondary metabolites, a family of mycotoxins affecting animal and human health [5]. Disease control by chemical and agronomic approaches is often ineffective and increases the cost of production [6]. For this reason host resistance is the most durable and sustainable method to reduce losses.

Several studies reveal that genetic variation for *Fusarium* ear rot resistance and fumonisin contamination exists among hybrids and inbred lines of field maize [7, 8]. However, there is no evidence of complete resistance to either trait in maize. *Fusarium* ear rot resistance is under polygenic control and strongly influenced by environmental factors [9]. The complexity of this trait has hampered breeding for resistance, therefore most commercial maize hybrids are susceptible [10]. However, screening trials performed for both *Fusarium* ear rot and fumonisin contamination using public and private inbred lines identified several lines and hybrids with good levels of resistance to both *Fusarium* ear rot and fumonisin accumulation [11].

Linkage-based mapping studies in biparental populations have shown that quantitative trait loci (QTL) to *Fusarium* ear rot resistance have relatively small effects and are not consistent between populations [12–15]. We previously constructed a genetic linkage map by using single nucleotide polymorphism (SNP) markers, generated from genotyping by sequencing (GBS) technology [Marocco, unpublished data]. Markers were developed on the segregating population derived from the cross between CO441 and CO354 maize lines, previously classified as resistant and susceptible to *Fusarium* ear rot [11, 16, 17]. Progress in breeding for resistance will be speeded up by analysis of new and consistent QTLs for *Fusarium* ear rot resistance and fumonisin accumulation and by a deeper knowledge of genetic mechanisms underlying maize-*F. verticillioides* interactions.

Large studies on model species have clarified some crucial events in plant resistance. Plants have evolved two defense mechanisms to resist pathogen invasion that involve different strategies of detecting pathogens. On the extracellular face of the host cell, pathogen associated molecular patterns (PAMPs) are recognized by pattern recognition receptors (PPRs) and the subsequent stimulation of PPRs leads to PAMP-triggered immunity (PTI) [18]. Its induction leads to mitogen activated protein kinases (MAPKs) and calcium signaling, transcription of pathogen-responsive genes, production of reactive oxygen species (ROS) and deposition of callose to reinforce the cell wall at sites of infection [19]. Furthermore, plants have evolved a more specialized defense mechanism towards successful pathogens, the effector-triggered immunity (ETI), which acts largely inside the cell and involves the recognition of pathogen delivered effectors contributing to pathogen virulence by plant resistance (R) proteins. ETI is an accelerated and amplified PTI response, resulting in disease resistance and, usually, a hypersensitive cell death response (HR) at the infection site. Following the early signaling events activated by pathogen attack, elicitor signals are often amplified through the generation of secondary signal molecules, such as salicylic acid (SA), ethylene (ET) and jasmonates (JA). However, cross talk and synergistic effects between the defense pathways mediated by SA and JA/ET during different pathogenic infections has been proposed [20]. Furthermore, in several plant-pathogen interactions, the defense response has been associated with the accumulation of flavonoids, phenolic compounds, and phytoalexins [21]. In maize, upon fungal inoculation, induction of 3-deoxyanthocyanidin flavonoids was observed in silks and kernels of resistant lines, suggesting a role for this compound in resistance to *F. verticillioide*s [21]. In addition, elevation of phenylpropanoids in the maize kernel pericarp is a trait associated with lower disease severity and fumonisin accumulation caused by *F. verticillioide*s [22].

Microarray analysis was previously applied to characterize *F. verticillioides*-maize interaction [16, 17]. We reported that defense responses involved changes in the expression of a large number of genes in seeds of susceptible (CO354) and resistant (CO441) maize genotypes. However, in the resistant line, the expression of defense-related genes was observed before inoculation, while in the susceptible genotype defense genes were induced only after pathogen attack. The study was extended to the analysis of enzymes involved in removal of ROS, namely ascorbate peroxidase (APX), catalase (CAT), peroxidase (POX) and superoxide dismutase (SOD) [17]. In resistant seedlings, before infection, APX and SOD enzymatic activities were higher compared to the susceptible ones, while after 5 days, they remained unchanged. On the other hand, in the susceptible seedlings all enzymes assayed were activated only after *F. verticillioides* inoculation. These findings supported the hypothesis of a basal defense response provided by the resistant genotype in maize both in kernels and seedlings.

RNA-Seq is increasingly being used for global gene expression profiling in plants [23, 24] as it provides significant advantages over traditional microarray analysis: e.g. accurate absolute quantification of expression data, high sensitivity and reproducibility for both technical and biological replicates, broader capability of detecting differential expression over a large dynamic range as well as the possibility of detecting novel expressed genes [25]. Recent studies have described the RNA-Seq technology use in maize [26, 27], nevertheless this approach has never been used to compare defense responses to pathogens in resistant vs. susceptible maize genotypes.

In this work, RNA-Seq was used to identify the early transcriptional changes in both resistant CO441 and susceptible CO354 genotypes responding to *F. verticillioides* inoculation. It was observed that CO441 response was characterized not only by a constitutive expression, but also by a prompt and enhanced induction of some key genes associated with resistance mechanisms, antioxidant process, hormone signaling, signal transduction cascades and secondary metabolism. These findings will provide valuable candidate genes that could be used to develop *Fusarium* ear rot-resistant maize genotypes.

## Results

### *F. verticillioides*growth on CO441 and CO354 maize genotypes

The two maize inbreds CO441 and CO354, previously classified as resistant and susceptible to *F. verticillioides* according to their field behavior and growth assay by absolute quantification of the fungal *β-tubulin2* (*TUB2*) transcript through real-time RT-PCR analysis, were used in this study [11, 16, 17]. Infection progression in the area surrounding *F. verticillioides* inoculated kernels was monitored in the early stages of infection over a time course of 96 hours. The *TUB2* transcript was detected in kernels of susceptible and resistant genotypes at 48 through 96 hours post inoculation (hpi). The highest transcript copy number was found at 96 hpi in both genotypes, although in the resistant line CO441 gene copy number was about twenty-nine times lower compared to the susceptible line CO354 (99.8 ± 16.1 vs. 2,856 ± 250, respectively; Table 1). This profile was indicative of stronger resistance mechanisms in CO441 genotype, still active at 72 hpi, and for this reason 72 hpi was chosen as the relevant time-point to perform RNA-Seq analysis.

**Table 1:** **Analysis of** ***F. verticillioides*** **inoculation steps**

	12 hpi ^1^		24 hpi		48 hpi		72 hpi		96 hpi	
	Copy N° ^2^	sd ^3^	Copy N°	sd	Copy N°	sd	Copy N°	sd	Copy N°	sd
**CO441 INOC.** ^**4**^	0	0	0	0	14.28	1.5	22.71	1.3	99.8	16.1
**CO354 INOC.**	0	0	0	0	77.71	12	284.70	50	2,856	250

^1^hpi = hours post inoculation; ^2^Copy N° = Copy number; ^3^sd = standard deviation; ^4^INOC. = inoculated.

Copy number of transcripts for the constitutive gene *β-tubulin 2* in kernels of resistant CO441 and susceptible CO354 maize lines inoculated with the fungus, over a time course of 96 hours.

### Transcriptome sequencing of uninoculated and *F. verticillioides*inoculated CO441 and CO354 maize kernels

**Figure 1:**
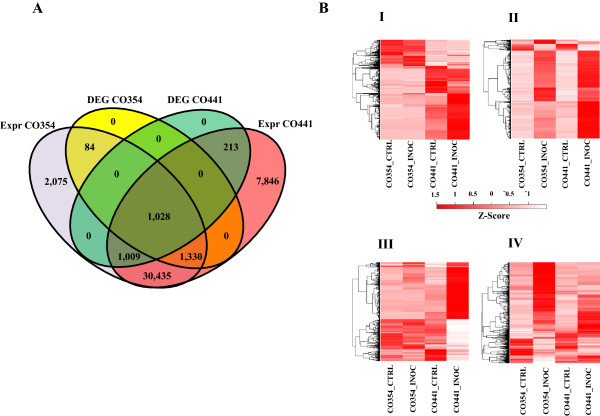
**Expressed and differentially expressed genes before and after** ***F. verticillioides*** **inoculation. (A)** Venn diagrams of total and *F. verticillioides*-inoculated genes. Venn diagrams illustrating the relationships between total expressed (expr) genes and differentially expressed genes (DEGs) after *F. verticillioides* inoculation in the two maize genotypes CO354 and CO441. **(B)** Clustering and heat maps of differentially expressed genes grouped into 4 clusters based on the expression profiles. Modulated genes are distinguished in genes differentially regulated between CO354 and CO441 genotypes before *F. verticillioides* inoculation (Cluster I), genes modulated by *F. verticillioides* common to both genotypes (Cluster II), genes specific to CO441 and CO354 lines (Cluster III and IV, respectively). The colour scale indicates the FPKM expression values (darker red indicate higher level expression values, light red indicates lower gene expression values). The heat map and clustering of FPKM expression values were generated with custom scripts using Euclidean distance measure with average linkage.

Transcriptome sequencing of libraries obtained from resistant and susceptible maize kernels either uninoculated or inoculated produced 197,930,844 and 235,394,377 paired-end 100 bp reads, respectively, corresponding to about 39.6 Gbps and 44.9 Gbps (Additional file 1: Table S1). On average 74% of the total reads mapped to maize inbred B73 reference genome sequence (Additional file 1: Table S1), with most of the reads mapping to exons (~96%) and only a small proportion mapping to introns (~1.8%) and intergenic regions (~2%; Additional file 2: Figure S1). Gene expression levels were quantified by using the maize B73 as reference genome and the abundance of each transcript was expressed as fragments per kilobase of exon model per million mapped reads (FPKM) as implemented by Cufflinks [28]. We detected 41,861 and 35,961 known protein coding genes (ZmB73_5a working gene set) expressed at a FPKM level > 0.1 in the CO441 and CO354 genotypes, respectively, out of which ~75% in the filtered gene set (ZmB73_5b) (Additional file 3: Table S2; Figure 1A). In addition, 2,728 and 2,220 putative novel expressed loci were found in each of these genotypes, representing potentially important new information for genome annotation (Additional file 3: Table S2). Raw counts expression data showed a Pearson’s correlation among biological replicates >0.99 for all the samples analyzed, indicating high correlation between sequencing replicates.

Expression profiles of inoculated CO441 and CO354 were compared with the respective uninoculated controls, while basal differences were investigated by comparing uninoculated CO441 and CO354 samples. Genes were defined as differentially expressed genes (DEGs) with a False Discovery Rate (FDR) threshold of 0.05 and a Log2 fold-change (FC) ≥1 [29]. Transcriptome analysis revealed a total number of 6,951 DEGs, grouped into 4 clusters based on their expression profiles (Figure 1B). To gain insights into the functionality of the genes with constitutive differences and responsive to *F. verticillioides* inoculation DEGs were annotated with Blast2GO software [30] and classified in 13 functional categories.

### Intergenotypes differences in basal gene expression

Basal level differences between the resistant and susceptible genotypes were explored by comparing uninoculated CO441 and CO354 control samples (Cluster I, Figure 1B). Differential expression analysis revealed 2,551 significantly DEGs (Additional file 4: Table S3), 73% of which were more abundant in the resistant genotype. As the higher resistance of CO441 inbred line to *F. verticillioides* might reflect the presence of stronger constitutive physical or chemical barriers [31], we focused on genes belonging to defense-related functional classes, as resistance, response to stress, cell wall and secondary metabolism. In particular we found that 86% of DEGs belonging to secondary metabolism functional class were up-regulated in CO441 line and down-regulated in CO354 line. Moreover, out of 2,551 DEGs between the genotypes, 302 genes expressed at low level in CO354 (FPKM <0.5) had a much higher expression value in CO441, ranging from 5 FPKM to ~3692 FPKM. On the contrary, only 57 genes expressed at low level in CO441 (FPKM <0.5) were expressed more than 5 FPKM in CO354. In particular, by applying these criteria, the two functional categories secondary metabolism and transport displayed only CO441-specific genes (Table 2; Additional file 4: Table S3).

**Table 2:** **Comparison between differentially expressed genes in uninoculated CO441 and CO354 genotypes**

Functional category	N° genes	
	FPKM_CTRL CO441 > 5; 0 ≤ FPKM_CTRL CO354 < 0.5	FPKM_CTRL CO354 > 5; 0 ≤ FPKM_CTRL CO441 < 0.5
**All defense-related genes**	29	7
*Cell wall*	1	4
*Resistance*	5	1
*Response to stress*	12	2
*Secondary metabolism*	11	/
**Cell component**	9	2
**Electron/energy**	8	1
**Metabolic process**	90	17
**Miscellanea**	20	2
**Photosynthesis**	1	1
**Proteolysis**	4	2
**Signal transduction**	31	4
**Transport**	9	/
**Unknown function**	101	21

The table shows the total number of genes whose basal expression is higher in the resistant CO441 or susceptible CO354 genotype within each category, considering the following filters: FPKM >5 in CO441 and 0 ≤ FPKM <0.5 in CO354, and FPKM > 5 in CO354 and 0 ≤ FPKM < 0.5 in CO441, respectively. Defense-related genes considered for the comparison are those functionally categorized as ‘cell wall’, ‘resistance’, ‘response to stress’ and ‘secondary metabolism’.

Overall, these data show that DEGs belonging to all functional classes were mainly over-expressed in CO441 genotype compared to CO354 at basal level, and in particular for the secondary metabolism category, suggesting that the resistance to *F. verticillioides* infection in this genotype could be potentially related to the constitutive expression of secondary metabolism-related genes (Table 2; Additional file 5: Figure S2).

### Transcriptional changes in response to *F. verticillioides*inoculation

**Figure 2:**
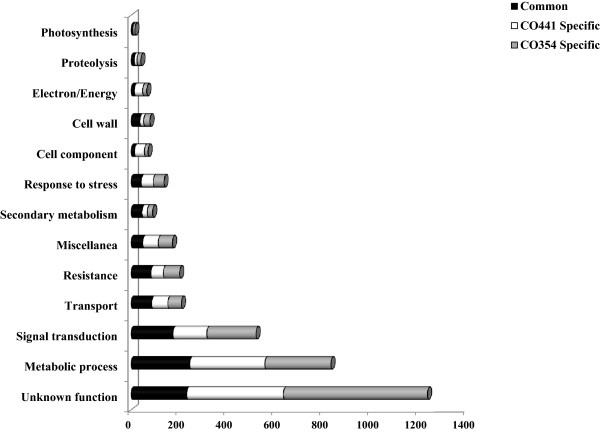
**Specificity of transcriptional changes in inoculated CO441 and CO354 genotypes within functional categories.** Proportion of genes modulated in CO441 (white) and CO354 (light grey) or in both genotypes (black) at 72 hpi.

Transcriptional changes induced by *F. verticillioides* in susceptible and resistant genotypes were determined by comparing inoculated samples at 72 hpi with uninoculated control samples and DEGs are shown in Figure 1 (full list provided in Additional file 6: Table S4). A total of 2,250 (1,656 induced, 594 repressed) and 2,442 (2,024 induced, 418 repressed) genes distributed in the different functional classes were found as differentially regulated after *F. verticillioides* inoculation in resistant and susceptible genotypes, respectively (Additional file 7: Figure S3). In both lines, the majority of modulated transcripts was up-regulated, although in the susceptible genotype a higher percentage of pathogen-induced genes was detected (~73% vs. ~83% for CO441 and CO354, respectively). The amount of genes for each functional category whose modulation in response to inoculation was common to both genotypes, or restricted to one or the other, is reported in Figure 2. Although genotype-specific transcriptional changes were found, genes modulated in both genotypes represent an important proportion (28%) in nearly all different functional classes, indicating a certain level of conservation in pathogen response.

#### Common transcriptional changes in response to inoculation

**Figure 3:**
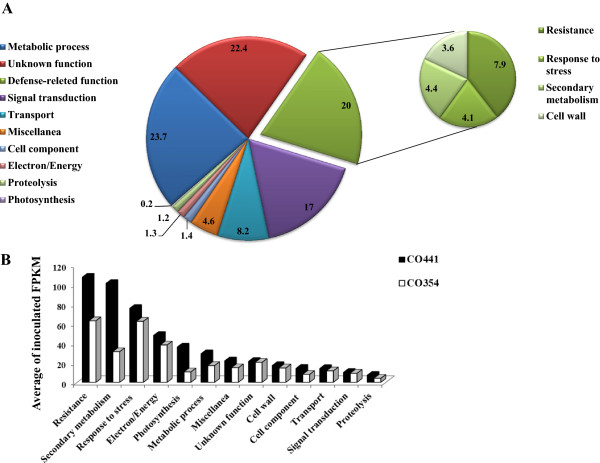
**Common transcriptional changes in CO441 and CO354 at 72 hpi. (A)** Distribution of 1,028 common genes, modulated in both genotypes at 72 hpi into functional categories. Defense-related category was detailed in a smaller pie on the right. **(B)** Average of FPKM values of common genes in CO441 (black) and CO354 (white) genotypes at 72 hpi.

A total of 1,028 DEGs were co-modulated in both genotypes. After discarding genes with unassigned function, the largest proportion of common genes was related to metabolic process (23.7%), followed by genes involved in disease resistance (20%), that included the categories: resistance (7.9%), response to stress (4.1%), secondary metabolism (4.4%), and cell wall (3.6%) (Figure 3A). This class consisted of several modulated genes encoding chitinases, serine-protease inhibitors, glutathione S-transferases (GST) and terpene synthases. Although they were differentially regulated in both genotypes, important differences in the level of induction were especially notable for these genes (Additional file 6: Table S4). Beside the disease resistance category, the next largest group of common transcripts was related to signal transduction (17%, Figure 3A). This group included many genes encoding WRKY, NAC and MYB transcription factors (TFs), which were much more strongly induced in CO441 (inoculated FPKM values ranging from about 0.6-72 in the resistant genotype; 0.1-28 in the susceptible genotype). In line with this, if we considered the average of inoculated FPKM values for each functional category, expression levels of common DEGs were found invariably much higher in CO441 and the three classes with the higher average FPKM values were: resistance, secondary metabolism and response to stress (Figure 3B). By visualizing common DEGs in both genotypes related to biotic stress processes again a response with higher magnitude was detected in CO441 compared to CO354 genotype in nearly all considered categories (Additional file 8: Figure S4). Therefore, even if a certain number of genes was found to be modulated by the *F. verticillioides* in both genotypes, an enhanced response was observed in the resistant genotype mainly affecting defense-related genes.

#### Genotype-specific transcriptional changes in response to inoculation

**Figure 4:**
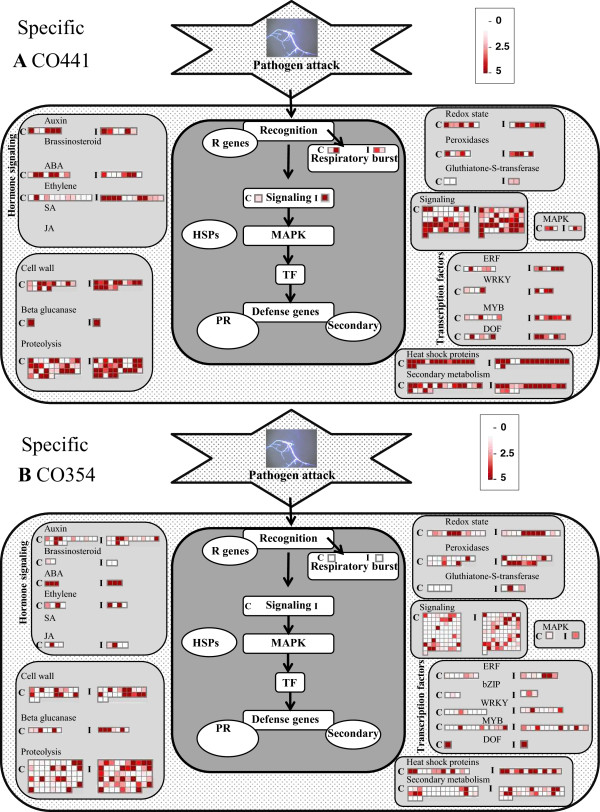
**Distribution of differentially expressed genes specific to CO441 and CO354 genotypes related to biotic stress processes, visualized by MapMan.** Each square represents the FPKM expression value for one gene in control (heatmap on the left within each category) and inoculated (heatmap on the right within each category) resistant CO441 **(A)** and susceptible CO354 **(B)** genotypes.

Specific transcriptional changes induced by *F. verticillioides* included 1,222 and 1,414 genes found as differentially regulated in CO441 and CO354 genotypes, respectively (Figure 1A and B; Additional file 6: Table S4). The most prevalent functional categories among the modulated genes were metabolic process (25.6% and 19.4%, respectively) and signal transduction (11.5% and 14.5%, respectively) for both maize lines (Figure 2). The category of defense-related genes followed with a higher percentage of DEGs for the resistant genotype (15.2% and 10.2% for CO441 and CO354, respectively). These percentages were lower in comparison to the percentage of defense-related DEGs found in common transcriptional changes (20%; Figure 3A), suggesting that most of plant responses to pathogen were in common to CO441 and CO354 lines, although with an enhanced reaction in the resistant genotype (Figure 3B). In addition, average FPKM values in inoculated kernels for both genotype-specific DEGs decreased compared to common transcriptional changes. However higher average FPKM values upon inoculation were observed in CO441 compared to CO354, in particular for defense-related categories, where the inoculated resistant line showed, in average, FPKM expression values of ~28, against FPKM values of ~19 in the susceptible genotype. The highest difference was observed again in secondary metabolism category (~15 vs. ~1.5 FPKM expression values in inoculated CO441 and CO354 samples in average, respectively). Specific genes involved in shikimate biosynthesis (e.g. shikimate kinase, GRMZM2G161566), phenylpropanoid biosynthesis (e.g. 4-coumarate-ligase, GRMZM2G096020) and lignin biosynthesis pathways (e.g. cinnamyl-alcohol dehydrogenase, GRMZM2G090980) appeared to be strongly influenced by inoculation in CO441 with up to ~12-fold induction. Response to stress and resistance categories followed, as for common DEGs, where average FPKM expression values were 2–3 higher in the resistant genotype compared to the susceptible one. By visualizing transcriptional changes related to biotic stress processes, the same trend was observed and higher FPKM values were revealed in inoculated CO441 sample compared to CO354 (Figure 4A and B). Even though all these genes were well represented in both genotypes, it was observed that on the whole CO441, beside presenting higher basal expression level for many of these genes, shows an enhanced gene induction, confirming its strengthening also in the specific response to inoculation (Figure 4A; Additional file 6: Table S4).

### Validation of RNA-Seq data by real-time RT-PCR

To validate the RNA-Seq expression profiles of DEGs real-time RT-PCR was performed on 33 genes randomly selected for high or low FPKM expression levels. The comparison between the two techniques revealed a substantial agreement for all nine genes differentially expressed at basal level between CO441 and CO354 genotypes (Additional file 9: Figure S5). Generally, as predicted, the resistant genotype had higher expression levels before inoculation for all assayed genes, although RNA-Seq analysis displayed a higher dynamic range, showing larger differences between FPKM values compared to real-time RT-PCR.

Real-time RT-PCR on twenty-four DEGs previously identified comparing inoculated plants with their respective controls and including both genes modulated in both genotypes and genotype-specific DEGs showed expression profiles consistent with the RNA-Seq data for twenty genes (Additional file 10: Figure S6A-T). The enhancement in expression levels and defense response revealed by RNA-Seq results in the resistant genotype was confirmed by the real-time RT-PCR, in line with an enhanced *Fusarium* responsiveness at 72 hpi in this genotype.

### Identification of maize novel genes in response to *F. verticillioides*inoculation

By mapping RNA-Seq reads against the maize B73 reference genome sequence, only a small portion of reads matched to intergenic regions, from which 4,948 putative novel expressed loci were obtained in the CO441 and CO354 genotypes (Additional file 2: Figure S1; Additional file 3: Table S2). To determine whether these genes encoded proteins, the coding potential of each contig was estimated using Coding Potential Calculator (CPC) [32]. CPC indicated that ~13.5% and ~4.9% of these novel expressed genes had a coding and a highly coding potential, respectively. As for known genes, novel genes with statistically significant differential expression were determined, resulting in 132 and 156 novel genes specifically regulated in CO441 and CO354, respectively, and only 10 novel genes regulated in both genotypes by *F. verticillioides* inoculation (Additional file 6: Table S4). Functional annotations revealed that although more than 80% of differentially expressed novel genes were uncharacterized proteins, 8% and 5% of total novel genes belonged to the categories metabolic process and signal transduction, respectively (Additional file 6: Table S4). Interestingly, among differentially expressed novel genes an o-glycosyl hydrolase family 17 protein (XLOC_029870) induced by pathogen in the susceptible genotype showed a high coding potential. The alignment of XLOC_029870 and its corresponding transcript TCONS_00067999 were associated to annotated gene GRMZ5G846916 (Additional file 11: Figure S7A). Analyzing the protein sequence deriving from the gene GRMZ5G846916, we found that the annotated gene encoded a glycosyl hydrolase protein of 246 amino acids (IPR000490). On the other hand, the novel gene XLOC_029870 encoded a 649-amino acids protein showing at the C terminus region the presence of catalytic domains ×8 (IPR000490, IPR012946), possibly involved in cellulose binding, and lacking in the annotated gene GRMZ5G846916 (Additional file 11: Figure S7B) [33]. Semi-quantitative RT-PCR confirmed the RNA-Seq identified transcript, suggesting that RNA-Seq method not only identifies novel genes, but also improves the annotation of incomplete or partially annotated known genes (Additional file 12: Figure S8).

## Discussion

In order to extend our knowledge about the molecular changes produced by infection with one of the most important maize pathogens, *F. verticillioides*, we analysed differential gene expression induced by this pathogen using RNA-Seq technology in resistant and susceptible maize inbreds. Although some of the mechanisms affecting disease resistance in earlier stages were evaluated in previous works by microarrays, this study reports on the first use of RNA-Seq related to the pathosystem *F. verticillioides*-maize.

### Expression of plant resistance genes

Plants have evolved and adapted numerous defense mechanisms in response to a wide variety of pathogens. Pathogen recognition involves two kinds of receptors: those located in the plasma membrane and those present in the cytoplasm. Receptors located in the plasma membrane recognize conserved pathogen- or microbe-associated molecular patterns (PAMPs or MAMPs) and belong to families of receptor-like kinases (RLKs) [34, 35]. In our study RLK-leucine-rich repeat (LRR) protein kinases showing higher FPKM values in the CO441 control kernels compared to CO354 were identified, some of which retained similar FPKM values after *F. verticillioides* inoculation. Additionally, some RLK-LRR kinases were up or down regulated upon inoculation either in both genotypes or specifically in one of them. In any case a stronger pathogen induced modulation was found in CO441 and also RLK-LRR specifically induced in the susceptible genotype showed higher FPKM values in CO441 than in CO354 upon inoculation.

Two genes encoding brassinosteroid insensitive 1-associated receptor kinase 1 (BAK1) precursor, a LLR-RLK protein known to be associated with the receptor kinase flagellin-sensitive 2 (FLS2) and required to mediate PAMP induced immunity [36], were induced by pathogen in both genotypes. Interestingly a BAK1 precursor was found specifically up-regulated in the resistant genotype (2-fold induction), but not in the susceptible one (AC217401.3_FG003). These results suggest that a difference in surveillance system exists between genotypes already at basal level. Furthermore, even at 72 hpi some resistance-related receptors still retain activation capability especially in CO441 genotype. This is not surprising if we consider that sampling included kernels surrounding the inoculation point. Nucleotide binding site (NBS)-LRR proteins encoding for R (resistance) genes act as the second layer of active plant immunity in the cytosol [37]. A number of NBS-LRR encoding transcripts were also found among DEGs, with similarity mainly to RPM1 or RPP13, presenting an analogous induction pattern as RLK-LRR. This indicates therefore the possible involvement of R genes at some stages of this interaction.

Downstream signaling networks are triggered by pathogen recognition and mediated by protein kinases (PK), especially calcium-activated PKs (such as CIPKs or CDPK) and MAPK, that control defense responses [38–40]. Consistent with this widely accepted model, we detected an huge array of genes encoding for PK modulated by the inoculation, as well as CIPK/CDPK, MAPK and SNF1-related PK. Interestingly, four CDPKs family members presented a strong modulation among the two genotypes (average FPKM values of 0.02 vs. 7.6 for the resistant and susceptible genotypes, respectively), and further additional CDPKs modulated their expression upon inoculation mainly in CO441.

Calcium homeostasis regulators (e.g. calmodulin, calmodulin-binding protein, calcium transporting ATPases, calcineurin B-like proteins) involved in defense [18] also changed their expression mainly in *F. verticillioides* CO441 inoculated kernels. In *Arabidopsis*, plants pathogen perception leads to the activation of two independent MAPK cascades, the MEKK1-MKK4/MKK5-MPK3MPK6 cascade and the MEKK1-MKK1/MKK2-MPK4 cascade [41, 42]. In this study, homologs of AtMKK4 and AtMKK2 were induced in both genotypes upon inoculation, and interestingly the AtMKK2 (GRMZM2G400470) was found more induced in the CO441 genotype, supporting its involvement in CO441-mediated resistance to *F. verticillioides*.

Finally, some GTP-signaling related genes were differentially regulated according to the RNA-Seq analysis. In particular, four of them showed a higher expression at basal level in the CO441 genotype, providing additional support to G-protein association with defense responses in maize in line with previous studies [43–45].

Beside genes encoding for proteins involved in signaling, a high number of genes representing most of the 17 different pathogenesis-related (PR) classes were clearly identified as induced upon infection often shared by both genotypes. Finally, among bowman-birk protease inhibitors, which are typically modulated by JA in plant defense to regulate both endogenous as well as pathogen protease activity [46], five serine-protease inhibitors were detected as strongly induced and presenting very high level of expression. Two of them were induced by the pathogen only in the resistant genotype, while the remaining were differentially regulated in both genotypes, but with different values (~1947 FPKM in CO441). These data confirm the relevant role of proteinase inhibitors by helping to arrest pathogen invasions inhibiting proteolysis and thereby limiting availability of amino acids for growth and multiplication of the pathogen, especially for the resistant genotype.

PAMPs and MAMPs recognition is known to induce rapid production of ROS, affecting cellular oxidation state. Induction of GST is a widely recognized marker for ROS accumulation during defense [47–49]. A suite of twenty-three GST genes were up-regulated at 72 hpi in both genotypes, but in a more pronounced way in CO441, beside five genes presenting higher FPKM values in the resistant control kernels. Similarly, genes belonging to ascorbate peroxidases families, thioredoxins and a glutathione reductase, all bearing antioxidant enzymatic activity, were also found strongly differentially expressed at basal level, suggesting differences in the enzymatic antioxidant system in the two genotypes and their ability to cope with oxidative stress. Accordingly, heat shock proteins (HSPs) were mainly induced in CO354 (in particular hsp70 and hsp90 with FPKM values of about 122 and 160, respectively). This is in agreement with previous results reported by Campos-Bermudez and co-workers [50], that also found strong induction in HSPs in a susceptible inbred inoculated with *F. verticillioides*.

### Differential modulation of multiple TF families

Down-stream defense-responsive genes are normally regulated positively or negatively by TFs, direct or indirect targets of various signal transduction pathways. Among TFs a pivotal role in biotic responses is played by WRKY genes which represent one of the most represented class of TF found among DEGs in this study. WRKY genes were induced in both genotypes in response to pathogen inoculation, but showed a stronger induction in CO441 genotype, possibly related to an enhanced defense response. Increased levels of WRKY mRNA, protein and DNA-binding activity were previously reported following infection with viruses, bacteria or oomycetes, by fungal elicitors, SA and wounding. Among these WRKY22/29 work downstream of MPK3/MPK6 in PTI-related signaling induced by flg22 [41]. In our study a WRKY22-like gene (GRMZM2G149683) resulted differentially expressed at basal level comparing the two genotypes and strongly induced by pathogen mainly in CO441. No induction of WRKY70, typically induced during gene-for-gene resistance and related to salicylic acid signaling, was observed. Furthermore, WRKY proteins are known to regulate subsequently activated secondary-defense response genes [51]. Among these WRKY33 controls phytoalexin gene expression [52, 53]. As similar trends for WRKY33 induction were observed, other WRKYs more induced in CO441 may possibly contribute here to the magnitude and promptness of diterpene phytoalexin biosynthetic genes responses.

Other strongly represented TF families in DEGs were MYB, NAC and AP2/ERF TFs. *Arabidopsis* NAC TFs may play a dual role in regulating both JA- and abscisic acid (ABA)-dependent responses and manipulate plant stress responses by activating other genes encoding R2R3-MYB TF, amylase, cold responsive protein, dehydration responsive proteins, GST, and late embryogenesis abundant (LEA) proteins [54, 55]. We observed about a 1.5-fold induction in average for all NAC genes as well as for GST and MYBs following *F. verticillioides* inoculation in CO441 kernels. *Arabidopsis* R2R3-MYB TF directly acts on the promoters of the flavonoid biosynthesis genes and it is placed at the downstream end of the signaling cascade for flavonol-specific gene activation in phenylpropanoid biosynthesis [56]. Accordingly, we observed differential expression, both at basal level or after inoculation, of genes encoding enzymes related to biosynthesis of flavonol and phenylpropanoid, as discussed in the following paragraphs.

### Changes in hormone signaling-related genes

Plant resistance to biotrophic/hemibiotrophic pathogens activated by gene for gene recognition is controlled largely by SA-mediated signaling pathways, while resistance to necrotrophic pathogens is mediated by the JA and ET signaling pathways [57]. In our study we did not observe a significant induction of SA-related genes in both genotypes, in line with necrotrophic process. At the same time, the activation of typical JA-responsive defense genes such as JA-induced protein, chitinases, lipoxygenases and PR10 was observed with an enhanced induction for the CO441 at 72 hpi, especially concerning chitinases.

Beside JA, also genes involved in ET signaling were strongly represented among DEGs. Most of ACC oxidase genes and ET responsive proteins resulted up-regulated in both inoculated resistant and susceptible kernels, but mainly (2-fold) in CO441, suggesting ET signalling involvement in *F. verticillioides*-maize pathosystem, especially in the response of resistant genotype. In agreement with these observations an induction in both genotypes, but more pronounced in CO441, was also revealed for AP2/ERF TFs, which are known to be responsive to hormones and in particular to ET during biotic interactions [58, 59]. Interestingly, a general difference at basal level was also found for genes related to this pathway, such as the ET receptor (GRMZM2G420801) with FPKM values of ~25 and 2.4 in CO441 and CO354, respectively. The results described here substantiate the JA and ET involvement in the development of resistance to *F. verticillioides* in maize kernels, with a limited role for specific gene for gene recognition and SA mediated resistance [57].

### Changes in secondary metabolism-related genes

In the current study 126 genes were identified belonging to the secondary metabolism category, differentially regulated both between genotypes and at 72 hpi. Selected genes encoding for enzymes involved in the most represented pathways are discussed and shown in Additional file 13: Table S5.

Five genes from the shikimate pathway were induced in the resistant genotype. In particular the genes coding 3-deoxy-d-arabino-heptulosonate 7-phosphate synthase (DAHPS), 3-dehydroquinate dehydratase (3DQDHT) and prephenate dehydratase (PDHT) presented a substantially differential expression between the genotypes, while the expression of genes for shikimate kinase (SK) and aspartate aminotransferase (AAT) were specifically induced after *F. verticillioides* inoculation in the resistant genotype. This suggests a strong constitutive difference in shikimate pathway between genotypes possibly contributing to the resistance.

In a previous study the induction of genes involved in the shikimate pathway and the accumulation of phenylalanine, tyrosine and shikimate products were also observed in maize leaves at 8 days post inoculation with *U. maydis* [60]. More in detail both enzyme activity and transcript level of phenylalanine ammonia-lyase (PAL), a crucial enzyme involved in the biosynthesis of phenolic metabolites of the phenylpropanoid class, comprising hydroxycinnamic acid (HCA) derivatives, lignans and flavonoids [61], were found as strongly induced in inoculated tissue at the same time-point [60]. Here we identified five different PAL transcripts differentially expressed presenting different expression trends. They were all responsive upon inoculation, but for three genes the induction was more elevated in the resistant genotype and two were highly expressed only in the CO441 line before induction. Phenylpropanoid-related genes, as cinnamate 4-hydroxylase (C4H) and 4-coumarate:CoA ligase (4CL), and genes involved in lignin and lignan synthesis were also consistently modulated upon inoculation and at higher extent in the resistant genotype. Furthermore, a significant higher basal expression of the caffeoyl-o-methyltransferase 1 (COMT) was observed in CO441, suggesting that a constitutive reinforcement of cell walls by lignin and/or other phenolic compounds could contribute to the lower susceptibility [62]. In agreement with our results, previous findings showed that high level of phenylpropanoids in maize kernel pericarp is a trait associated to less disease severity and fumonisin accumulation caused by *F. verticillioides* [22].

RNA-Seq data also revealed significant changes in genes belonging to the flavonoid pathway upon inoculation, especially in CO441. Flavonoid biosynthesis starts with the amino acid phenylalanine and the end products include anthocyanins, flavones/isoflavones and condensed tannins. More in detail, a previous study investigated silk and kernel resistance to *F. verticillioides* and *F. graminearum* in maize lines differing for the 3-deoxyanthocyanidins and related 3-deoxyflavonoid (flavan-4-ols) content [21]. It was observed that upon fungal inoculation the induction of 3-deoxyanthocyanidins took place in resistant lines, suggesting the role of these compounds in resistance to *F. verticillioides.* Notably, our results indicated that there was a general induction of transcripts coding for enzymes of the anthocyanin pathway in CO441 and CO354 kernels, with a stronger modulation in the resistant genotype. Furthermore anthocyanins accumulation was also reported in maize tissues infected with *U. maydis*, coinciding with the induction of leucoanthocyanidin dioxygenase (LDOX) gene expression [60].

Terpenoids (or isoprenoids) are another well-characterized family of inducible defense chemicals. Maize produces diterpenoid phytoalexins, termed kauralexins, which are relevant components of maize pathogen disease response [63]. Terpenoids are produced in plant cells via mevalonate pathway (MVA) and methylerythritol 4-phosphate (MEP) pathways. Interestingly, in this study six genes encoding enzymes from the MEP pathway were detected as induced by pathogen in both genotypes, although they exhibited an enhanced expression (2-fold higher) in CO441 compared to CO354 genotype. More in detail, the 1-deoxy-d-xylulose 5-phosphate reductoisomerase (DXR), a regulatory points of the MEP and MVA pathways previously observed to increase during mechanical wounding and application of defense elicitors (namely methylJA and SA) [64], presented an enhanced induction in the resistant genotype after inoculation, beside a constitutive over-expression compared to the susceptible CO354.

Of all the up-regulated genes identified in this study and related to secondary metabolism, the most highly expressed in the CO441 inoculated kernels was the terpene synthase (TPS; GRMZM2G028306), also confirmed by real-time RT-PCR analyses, with a FPKM value of ~1564. TPS encodes a (s)-beta-macrocarpene synthase, putatively associated with the biosynthesis of zealexin [65]. Previous microarray analysis revealed that *Tps6/Tps11* genes were highly expressed in *Z. mays* following inoculation with several fungal pathogens including *F. graminearum*, and maize lines silenced in *Tps6/Tps11*displayed increased susceptibility to *U. maydis* infection [63, 65–67]. Furthermore, the induction of ent-copalyl diphosphate synthase (AN2) gene, involved in the biosynthesis of kauralexin phytoalexin, was observed in both genotypes after fungal inoculation, but with a higher FPKM value for the CO441. The strong induction of genes related to diterpene phytoalexin biosynthesis potentiated in CO441 genotype, therefore, confirms that *F. verticillioides* triggers phytoalexin biosynthesis throughout the infection process, and that these metabolites are central to the *Z. mays* defense response.

## Conclusions

RNA-Seq approach was employed for the first time in this study to investigate the molecular events involved in the establishment of resistance against *F. verticillioides* in *Z. mays*. The work demonstrates that the global transcriptional analysis provided an exhaustive view of genes involved in pathogen recognition and signaling network, and controlling synthesis and activities of TFs, enzymes, phytohormones, phytoalexins and other secondary metabolites, which coordinately contribute to host resistance against *F. verticillioides*. In addition, the advantage of providing absolute quantification of expression is crucial for the interpretation of the defense response and to identify candidates genes for additional functional genomic analysis. Useful alleles of maize genes can be now isolated from a wide range of inbred lines. Furthermore, the development of allele-specific markers linked to candidate genes will allow to test for association with resistance to *F. verticillioides* ear rot and fumonisin accumulation. The markers will be added to an existing SNP map generated from GBS of a segregating population developed from the cross between the resistant CO441 and the susceptible CO354 lines. QTLs for resistance to both traits will be detected and they will form the basis for both in-depth understanding of the inheritance mechanism and marker-assisted selection (MAS) for *F. verticillioides* resistance in maize.

## Methods

### Plant material and *F. verticillioides*inoculation procedures

Two maize inbreds exhibiting resistance (CO441) and susceptibility (CO354) to *Fusarium* ear rot [11, 16, 17] were evaluated after inoculation with conidial suspensions of *F. verticillioides*. Both the lines were developed by the Eastern Cereal and Oilseed Research Centre, Agriculture and Agri-Food Canada (AAFC), Ottawa, Canada, and were maintained by sibling at the Institute of Agronomy in Piacenza. Seeds of the lines were planted in pots (40 cm diameter, 35 cm height) and 10 plants of each line were grown up. Before inoculation, pots were transferred to an environmentally controlled greenhouse with day-time and night-time conditions of 28°C and 20°C temperature, respectively, and a light regime of 16 h using lamps at intensity of 500 μmol m^-2^ s^-1^ (Master TLD 58 W/830, Royal Philips Electronics, Eindhoven, The Netherlands).

*F. verticillioides* inoculation was performed using the isolate ITEM 1744 (Institute of Sciences of Food Production, National Research Council, Bari, Italy), a high fumonisin producer strain, cultured as previously described by [16, 17]. Conidia were collected by rinsing plates with sterile water, scraping the agar surface with a scalpel and filtering the conidia suspension through sterile cloth. Spore suspension was adjusted to a final concentration of 3.5 × 10^6^ conidia/ml based on microscopic counts using a Bürker chamber. Maize ears were inoculated at 15 days after hand-pollination (DAP) using a side-needle inoculator. The inoculating device consists of three 250 mm-long needles mounted on a plastic handle. Pins were dipped in the conidial suspensions and the bar was pressed through the husks sideways and into the centre of the ear, penetrating the kernels to a depth of 5–10 mm. For the detection of *F. verticillioides*, seeds adjacent the inoculated kernels were collected at 12, 24, 48, 72 and 96 hpi to evaluate fungal growth and colonization and to avoid mechanical damage due to needle-prick (Additional file 14: Figure S9). For RNA-Seq analysis, seeds were collected at 72 hpi. Control seeds were sampled at the same inoculation time listed above and considered as uninoculated. Three pools of kernels for each time-point were prepared, where each pool derived from the mixing of kernels coming from three different maize ears.

### RNA isolation and library preparation

RNA was isolated using Trizol reagent (Invitrogen, Carlsbad, CA, USA) and then purified with the RNA Clean up protocol (Qiagen, Valencia, CA, USA), according to the manufacturer’s instructions. RNA quality and quantity were determined using a Nanodrop 2000 spectrophotometer (Thermo Scientific, Wilmington, DE).

Total RNA samples were assessed for quality using a RNA 6000 Nano Kit (Agilent, Wokingham, UK) and 2.5-μg aliquots were used to isolate poly(A) mRNA for the preparation of a non-directional Illumina RNA-Seq library using the TruSeq RNA Sample Prep Kit v2 (Illumina Inc., San Diego, CA, USA). The quality of the library was checked with a High Sensitivity DNA Kit (Agilent, Wokingham, UK). Libraries were sequenced with an Illumina HiSeq 1000 sequencer (Illumina) and 100-bp paired-end sequences were generated.

### Bioinformatic analysis

The reads were aligned to the maize B73 reference genome (ZmB73_RefGen_v2; http://www.maizesequence.org) using TopHat v2.0.6 [68], giving as parameters the derived mean read spacer size (110 bp) and its standard deviation (100 bp). Alignments were processed with Cufflinks 2.0.2 [28] to assemble transcript isoforms and quantify expression value as fragments per kilobase of exon model per million mapped reads (FPKM) of known and novel genes using maize working gene set as reference annotation (AGPv2; http://www.maizesequence.org) and to guide RABT assembly using default parameters. Differential expression analysis was performed with DESeq package [29], with FDR threshold of 0.05 and a Log2 FC ≥ 1. Sequences of differentially expressed genes were compared with NCBI non redundant (NR) database with a Blast E-value of 10^-3^ and were functionally annotated using Blast2GO [30] assigning a GO term and a metabolic pathway in the Kyoto Encyclopedia of Genes and Genomes (KEGG) to the query sequences. Sequences were classified into 13 functional categories (Cell component; Cell wall; Electron/Energy; Metabolic process; Miscellanea; Photosynthesis; Proteolysis; Response to stress; Resistance; Secondary metabolism; Signal transduction; Transport; Unknown function) based on GO annotation.

The coding potential estimation of each novel genes was calculated using CPC [32] against the Uniref90 protein database (2012-01-04) [69], with default parameters.

Clustering of FPKM expression values of genes differentially expressed genes was performed using Euclidean distance measure with complete linkage.

### Data deposition

Transcriptome sequence data from this article can be found in the National Center for Biotechnology Information (NCBI) Sequence Read Archive under accession numbers SRR1186847, SRR1186858, SRR1186859, SRR1186861, SRR1186862, SRR1186863, SRR1186864, SRR1186866, SRR1186867, SRR1186869, SRR1186870, and SRR1186871.

### Real-time RT-PCR expression analysis

Real-time RT-PCR experiments were performed on seeds collected at 72 hpi, as reported before, using the 2× iQ SYBR Green Supermix (Bio-Rad, Hercules, CA, USA) and the CFX-96 device (Bio-Rad).

A 1 μg sample of total RNA was used for cDNA synthesis following the iScript cDNA synthesis kit protocol (Bio-Rad). 20 ng of single strand cDNA determined by fluorimetric assay (Qubit, Invitrogen) were used for real-time RT-PCR. Relative quantitative analysis was performed under the following conditions: 95°C for 3 min and 44 cycles at 95°C 10 s, 60°C 25 s. A melting curve analysis, ranging from 60 to 95°C, was used to identify different amplicons, including non-specific products [16, 17]. Three technical replicates (within each biological replicate) were employed for each tested sample and template-free negative controls. Gene-specific primers were designed within consecutive exons, separated by an intron, using Primer3 software and their sequences are shown in Additional file 15: Table S6. Relative quantification was normalized to the housekeeping control gene (*β-actin)* and FC in gene expression was calculated using the 2^-ΔΔCt^ method [70]. To quantify the growth of *F. verticillioides,* the copy number of *β-tubulin2* (*TUB2*) transcript was detected using real-time RT-PCR in seeds collected at 12, 24, 48, 72 and 96 hpi, as reported before. The primer pairs BT F1 and BT R2 were designed within a conserved region positioned between nucleotides 11 and 73 of the *TUB2* sequence (NCBI GenBank Accession No. GQ915447.1; Additional file 15: Table S6). Gene-specificity of primer pair was previously tested on several strains of *F. verticillioides* and other fungal species, such as *F. proliferatum* and *F. subglutinans* by PCR amplification using the following conditions: an initial step at 95°C for 3 min followed by 35 cycles at 95°C for 40 s, 56.7°C for 40 s and 72°C for 40 s and finally 72°C for 10 min. The real-time RT-PCR thermal cycling conditions were reported as indicated above [16, 17]. The number of *β*-tubulin copies is related to ng of cDNA obtained from kernels tissues and determined based on the equation of the linear regression according to the technical manual (Bio-Rad). Fungal cDNA (20 ng) from *F. verticillioides* isolate ITEM 1744 was serially diluted [1:1, 1:5, 1:5^2^, 1:5^3^, 1:5^4^, 1:5^5^] in sterile water and 20 ng of each kernel cDNA sample was compared to the dilution standard curve to determine fungal cDNA copy number.

### Semi-quantitative RT-PCR amplification

The semi-quantitative RT-PCR reaction was conducted in a final reaction volume of 20 μL containing 20 ng of single strand cDNA, 0.3 μM of each primer, 1× Buffer containing 1.5 mM of MgCl_2_, 0.2 mM of dNTPs and 0.3 U μL^-1^ of Taq Polymerase (GeneSpin, Italy). The PCR amplification profile was: 3 min initial denaturation at 94°C, followed by 35 cycles of 95°C for 40 s, 57°C for 40 s, 72°C for 50 s, and a 10 min final extension at 72°C. The amplified PCR products were separated in 1% agarose gel and stained with 3 μL of SYBR Safe DNA gel stain (Invitrogen). The length of PCR products was determined by comparison with the gene marker Superladder-Low 100 bp Ladder (Thermo Scientific).

## Electronic supplementary material

Additional file 1: Table S1: RNA-Seq sequencing and read mapping of each biological replicate. Numbers of RNA-Seq reads mapping to the maize genome are reported for each biological replicate (numbered from 1 to 3) for control (CTRL) and inoculated (INOC.) samples in CO441 (R) and CO354 (S) genotypes, respectively. (DOCX 23 KB)

Additional file 2: Figure S1: Distribution of RNA-Seq reads within the maize genome. Percentages (%) of reads mapping to exons (yellow), introns (red), intergenic (green) and UTR (blue) regions. The mean number of sequenced reads for three biological replicates are presented for each treatment in control (CTRL) and inoculated (INOC.) susceptible and resistant genotypes (CO354 and CO441, respectively). Percentages (%) are calculated with respect to the total mapping reads. (PPTX 44 KB)

Additional file 3: Table S2: Expression levels of maize known predicted and novel genes. Gene expression values (FPKM) and their relative confidence interval (FPKM_low and FPKM_high) are reported for all (62,874) maize known predicted genes and novel genes (4,948) in control (CTRL) and inoculated (INOC.) susceptible and resistant genotypes (CO354 and CO441, respectively). Common and genotype-specific novel genes are reported in separate sheets for easier access. (XLSX 7 MB)

Additional file 4: Table S3: Expression levels and annotation results of differentially expressed genes comparing uninoculated CO441 and CO354 genotypes. Gene expression values (FPKM) are reported for differentially expressed genes (2,551) in control (CTRL) CO354 and CO441 plants. DEGs were identified using DESeq package using a False Discovery Rate (FDR) <0.05. The logarithm of fold changes and functional annotation are reported for each gene. (XLSX 305 KB)

Additional file 5: Figure S2: Clustering and heat map of differentially expressed genes comparing uninoculated CO441 and CO354 genotypes. Genes whose expression differed significantly and belonging to the functional category secondary metabolism are reported. The colour scale indicates the FPKM expression values (darker red indicate higher level expression values, light red indicates lower gene expression values). The heat map and clustering of FPKM expression values were generated with custom scripts using Euclidean distance measure with average linkage. (PPTX 67 KB)

Additional file 6: Table S4: Expression levels and annotation results of differentially expressed genes and novel genes comparing CO441 and CO354 genotypes after *F. verticillioides* inoculation. Gene expression values (FPKM) are reported for differentially expressed genes (3,664) and novel genes (298) in control (CTRL) and *F. verticillioides* inoculated (INOC.) CO354 and CO441 plants. DEGs and differentially expressed novel genes were identified using DESeq package with a False Discovery Rate (FDR) < 5%. The logarithm of fold changes and functional annotation are reported for each gene. Common and genotype-specific DEGs and differentially expressed novel genes are reported in separate sheets for easier access. (XLSX 568 KB)

Additional file 7: Figure S3: Functional categories of differentially expressed genes modulated in CO441 and CO354 after *F. verticillioides* inoculation. DEGs in CO441 (A) and CO354 (B) at 72 hpi were annotated by Blast2GO analysis and classified in functional categories on the basis of literature evaluation. Induced genes are represented in light gray, while repressed ones are in black. The total percentage of modulated transcripts within each category is shown next to each bar. The complete list of genes is available in Additional file 6: Table S4. (PPTX 68 KB)

Additional file 8: Figure S4: Distribution of common differentially expressed genes in CO441 and CO354 genotypes related to biotic stress processes, visualized by MapMan. Each square represents the FPKM expression value for one gene in control (heatmap on the left within each category) and inoculated (heatmap on the right within each category) resistant CO441 (A) and susceptible CO354 (B) genotypes. (PPTX 394 KB)

Additional file 9: Figure S5: Comparison of RNA-Seq and real-time RT-PCR analyses for basal gene expression validation. Expression profiles of (A) heat shock protein 1 (GRMZM2G437100), (B) apoptosis inhibitor 5-like (GRMZM2G059039), (C) ethylene receptor (GRMZM2G420801), (D) germin-like protein 8-14-like (GRMZM2G343974), (E) protein peroxin-4-like (GRMZM2G461533), (F) phenylalanine ammonia-lyase (GRMZM2G118345), (G) terpene synthase (GRMZM2G028306), (H) calcium-dependent protein kinase (GRMZM2G047479), (I) pdr-like abc transporter (GRMZM2G445961). Histograms and errors bars represent normalized values relative to actin and standard deviations of three biological replicates, assessed by real-time RT-PCR in control kernels of the CO441 resistant line compared to the CO354 susceptible one, respectively. FPKM values are represented by dotted lines as assessed by RNA-Seq analysis. (PPTX 127 KB)

Additional file 10: Figure S6: Comparison of RNA-Seq and real-time RT-PCR analyses after *F. verticillioides* inoculation. Expression profiles of (A) terpene synthase 5 (GRMZM2G127087), (B) thaumatin-like protein (GRMZM2G402631), (C) cysteine proteinase inhibitor (GRMZM2G012160), (D) pathogenesis-related maize seed protein (AC205274.3_FG001), (E) Chitinase 2-like (GRMZM2G358153), (F) glucosidase 5-like isoform (GRMZM2G055699), (G) cinnamoyl-reductase (GRMZM2G107076), (H) phenylalanine ammonia-lyase (GRMZM2G160541), (I) WRKY DNA-binding domain (GRMZM2G057116), (J) globulin-1 s allele precursor (GRMZM2G026703), (K) cinnamyl-alcohol dehydrogenase (GRMZM2G090980), (L) cell wall invertase (GRMZM2G119689), (M) phospholipase d (GRMZM2G140811), (N) beta-glucanase (GRMZM2G12503), (O) 4-diphosphocytidyl-2-c-methyl-d-erythritol kinase (GRMZM5G859195), (P) farnesyl pyrophosphate synthetase (GRMZM2G098569), (Q) lipoxygenase (GRMZM2G109056), (R) cysteine protease 1 (GRMZM2G073465), (S) lipoxygenase (GRMZM2G067225), (T) peroxisomal acyl-oxidase 1a (GRMZM5G864319), (U) o-methyltransferase zrp4 (GRMZM2G101735), (V) germin-like protein (GRMZM2G178817), (W) lipid binding protein (GRMZM2G155555), (X) 3-ketoacyl-thiolase peroxisomal-like (GRMZM2G169380). Dotted lines and histograms represent values expressed as fold change of transcript levels in the inoculated kernels with respect to the transcript levels in control maize samples for the CO441 and CO354 genotypes assessed by RNA-Seq and real-time RT-PCR analysis, respectively. The asterisk (*) means that the FC revealed by RNA-Seq method for that gene is not included among DEGs for one of the two genotypes. (PPTX 167 KB)

Additional file 11: Figure S7: (A) Visualization of XLOC_029870 novel gene. Association of the GRMZ5G846916 annotated gene (I) with the predicted transcript TCONS_00067999 (II). (B) InterProScan protein structure. The 246-amino acids glycosyl hydrolase family 17 protein (ID: IPR000490) encoded by GRMZ5G846916 (I) and the 649-amino acids glycosyl hydrolase family 17 protein (IDs: IPR000490, IPR012946) encoded by XLOC_029870 are reported (II). The protein ID IPR012946 corresponds to the ×8 catalytic domain, possibly involved in carbohydrate binding and lacking in the annotated gene GRMZ5G846916. (PPTX 689 KB)

Additional file 12: Figure S8: Semi-quantitative PCR expression profile of XLOC_029870 novel gene. The amplification product (193 bp) was reported for each biological replicate (numbered from 1 to 3) in CO354 genotype at 72 hpi. Primer forward was designed flanking the nucleotide region common to the gene GRMZ5G846916 and the predicted transcript TCONS_00067999, while reverse primer flanking the nucleotide region specific only to TCONS_00067999. L = 100 bp DNA ladder. (PDF 37 KB)

Additional file 13: Table S5: Selected genes coding for enzymes related to biosynthesis of secondary metabolites. For each gene is reported: the Map number associated to that specific enzymatic pathway, gene name, gene acronym, enzymatic code (EC) according to KEGG database, annotation ID, gene expression values (FPKM) in control (CTRL) and *F. verticillioides* inoculated (INOC) CO354 and CO441 plants. Genes called as DEGs are indicated and associated to the following clusters: Basal (B); Common (C); CO441 specific (CO441 S) and CO354 specific (CO354 S). (XLSX 16 KB)

Additional file 14: Figure S9: Ear of the susceptible maize line CO354 at 72 (A) and 96 (B) hpi with *F. verticillioides*. Arrows indicate examples of kernels sampled surrounding the inoculation points. (PPTX 131 KB)

Additional file 15: Table S6: Details of the real-time RT-PCR analysis. The file contains: the sequence ID of each gene analysed by real-time RT-PCR and the corresponding primer pairs used for the amplification (FOR = forward primer, REV = reverse primer). (XLS 32 KB)
