# Supplementary material for: Functional genomic analysis of constitutive and inducible defense responses to Fusarium verticillioides infection in maize genotypes with contrasting ear rot resistance
Source: BMC Genomics. 2014 Aug 25;15(1):710. doi: 10.1186/1471-2164-15-710 (PMC4153945; doi:10.1186/1471-2164-15-710)
Supplement: Supplementary file 1 — Additional file 1: Table S1: RNA-Seq sequencing and read mapping of each biological replicate. Numbers of RNA-Seq reads mapping to the maize genome are reported for each biological replicate (numbered from 1 to 3) for control (CTRL) and inoculated (INOC.) samples in CO441 (R) and CO354 (S) genotypes, respectively. (DOCX 23 KB) [file 12864_2014_6392_MOESM1_ESM.docx]

**Additional file 1: Table S1. RNA-Seq sequencing and read mapping of each biological replicate.** Numbers of RNA-Seq reads mapping to the maize genome are reported for each biological replicate (numbered from 1 to 3) for control (CTRL) and inoculated (INOC.) samples in CO441 (R) and CO354 (S) genotypes, respectively.

| Samples | No. of reads | No. of mapped reads^*^ |
| --- | --- | --- |
| R^a^ CTRL^b^ 1 | 40,085,459 | 27,235,915 |
| R CTRL 2 | 33,788,984 | 26,135,684 |
| R CTRL 3 | 30,482,380 | 23,097,230 |
| R INOC.^b^ 1 | 31,900,086 | 23,419,427 |
| R INOC. 2 | 34,575,577 | 23,202,882 |
| R INOC. 3 | 27,098,358 | 17,590,612 |
| S^a^ CTRL 1 | 42,523,867 | 34,250,234 |
| S CTRL 2 | 32,173,317 | 25,859,325 |
| S CTRL 3 | 40,347,674 | 32,185,072 |
| S INOC. 1 | 41,848,656 | 31,281,328 |
| S INOC. 2 | 41,373,053 | 31,531,332 |
| S INOC. 3 | 37,127,810 | 27,218,834 |

^a^R= CO441; S= CO354. ^b^CTRL= Control, uninoculated; INOC.= Inoculated.

^*^Mapped on the maize B73 reference genome (ZmB73_RefGen_v2; http://www.maizesequence.org version 5b.60)
