# Supplementary figures and images for: Functional genomic analysis of constitutive and inducible defense responses to Fusarium verticillioides infection in maize genotypes with contrasting ear rot resistance
Source: BMC Genomics. 2014 Aug 25;15(1):710. doi: 10.1186/1471-2164-15-710 (PMC4153945; doi:10.1186/1471-2164-15-710)

## Slide 1
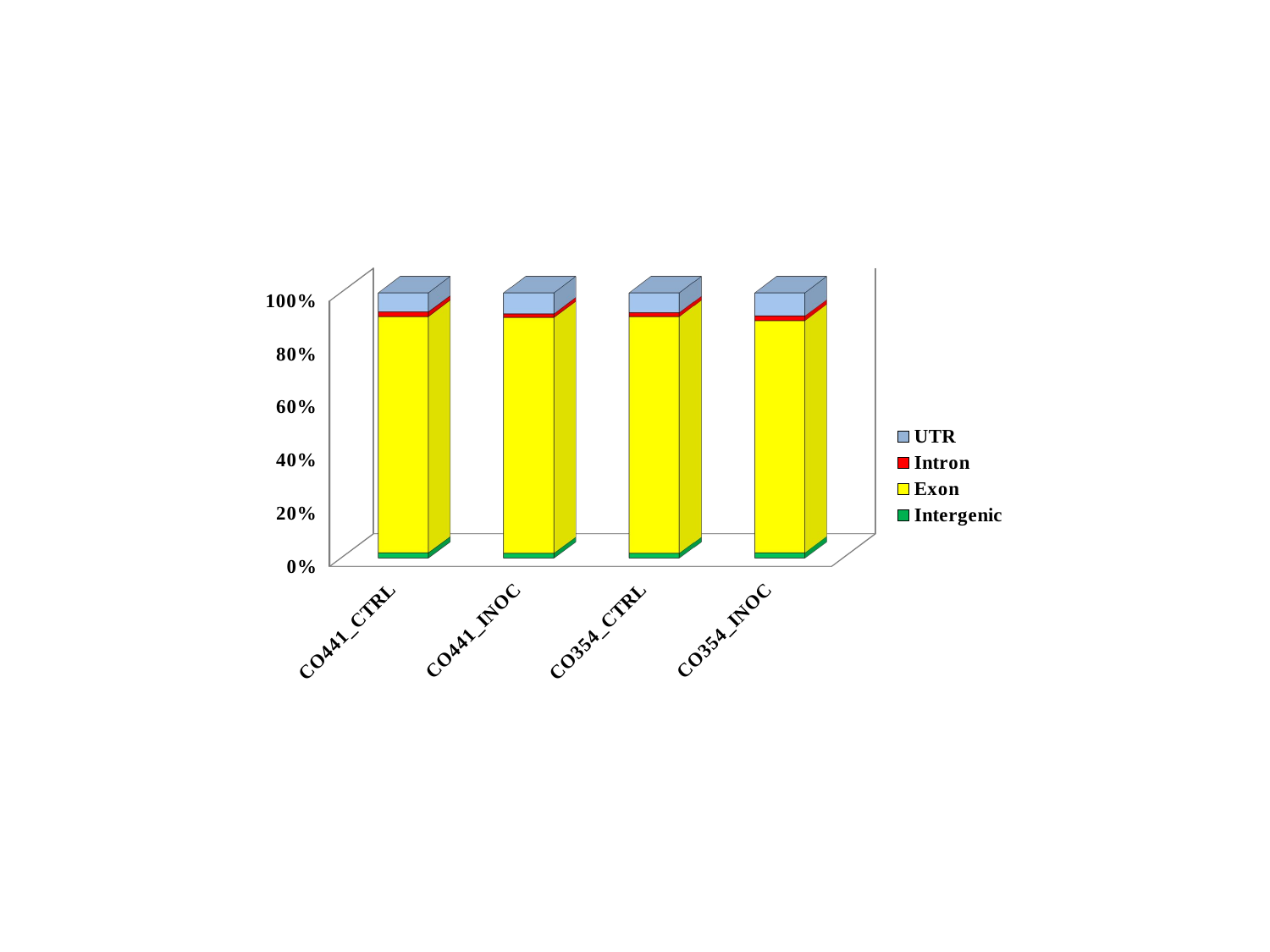

[unsupported chart]

Supplement: Supplementary file 2 — Additional file 2: Figure S1: Distribution of RNA-Seq reads within the maize genome. Percentages (%) of reads mapping to exons (yellow), introns (red), intergenic (green) and UTR (blue) regions. The mean number of sequenced reads for three biological replicates are presented for each treatment in control (CTRL) and inoculated (INOC.) susceptible and resistant genotypes (CO354 and CO441, respectively). Percentages (%) are calculated with respect to the total mapping reads. (PPTX 44 KB) [file 12864_2014_6392_MOESM2_ESM.pptx]

## Slide 1
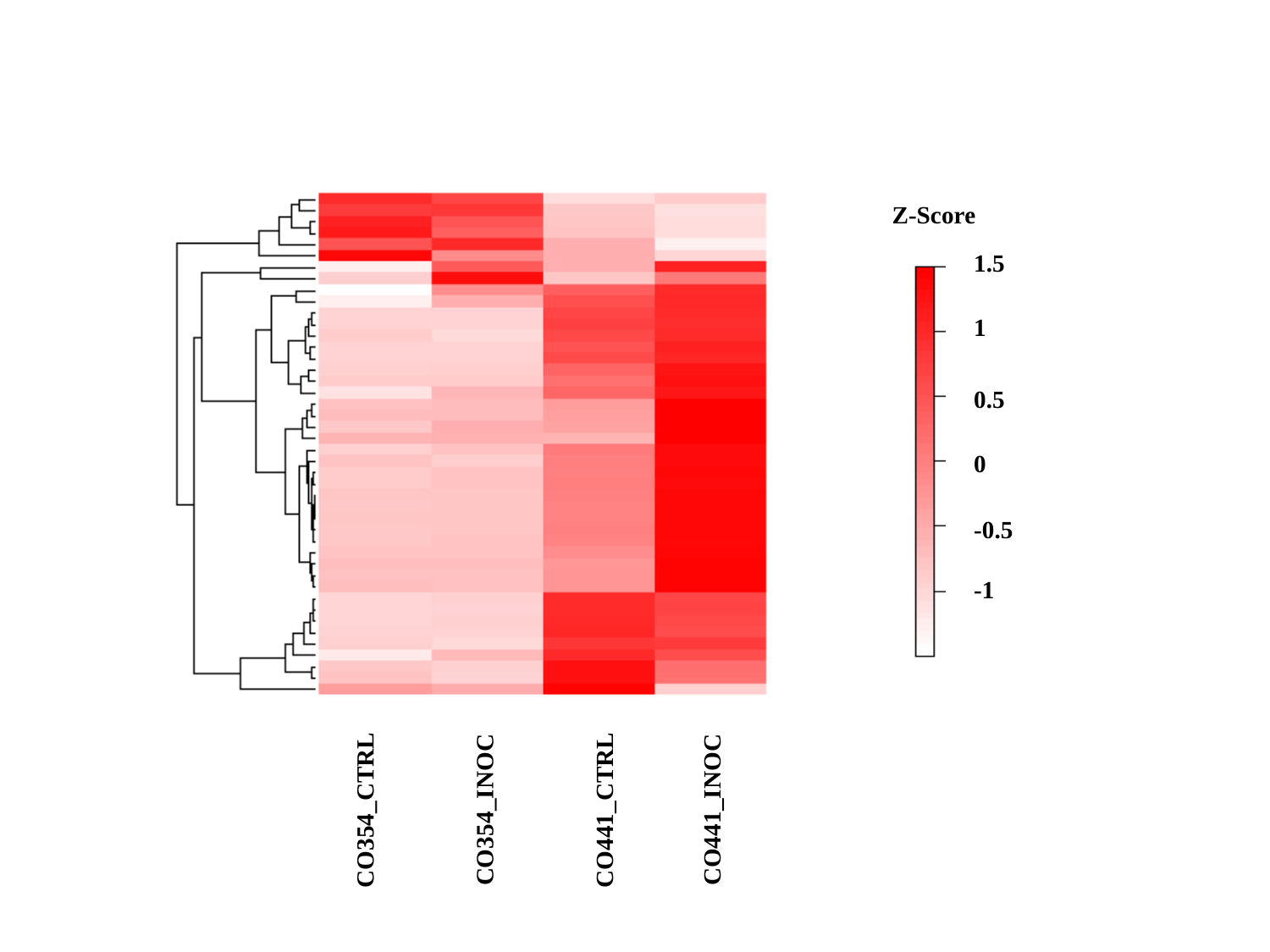

Z-Score
1.5
1
0.5
0
-0.5
-1
CO354_CTRL
CO354_INOC
CO441_CTRL
CO441_INOC

Supplement: Supplementary file 5 — Additional file 5: Figure S2: Clustering and heat map of differentially expressed genes comparing uninoculated CO441 and CO354 genotypes. Genes whose expression differed significantly and belonging to the functional category secondary metabolism are reported. The colour scale indicates the FPKM expression values (darker red indicate higher level expression values, light red indicates lower gene expression values). The heat map and clustering of FPKM expression values were generated with custom scripts using Euclidean distance measure with average linkage. (PPTX 67 KB) [file 12864_2014_6392_MOESM5_ESM.pptx]

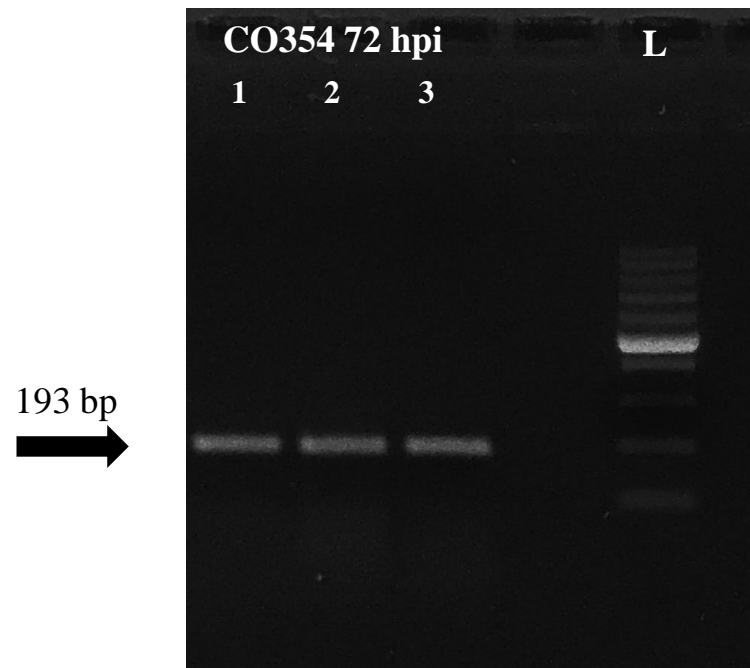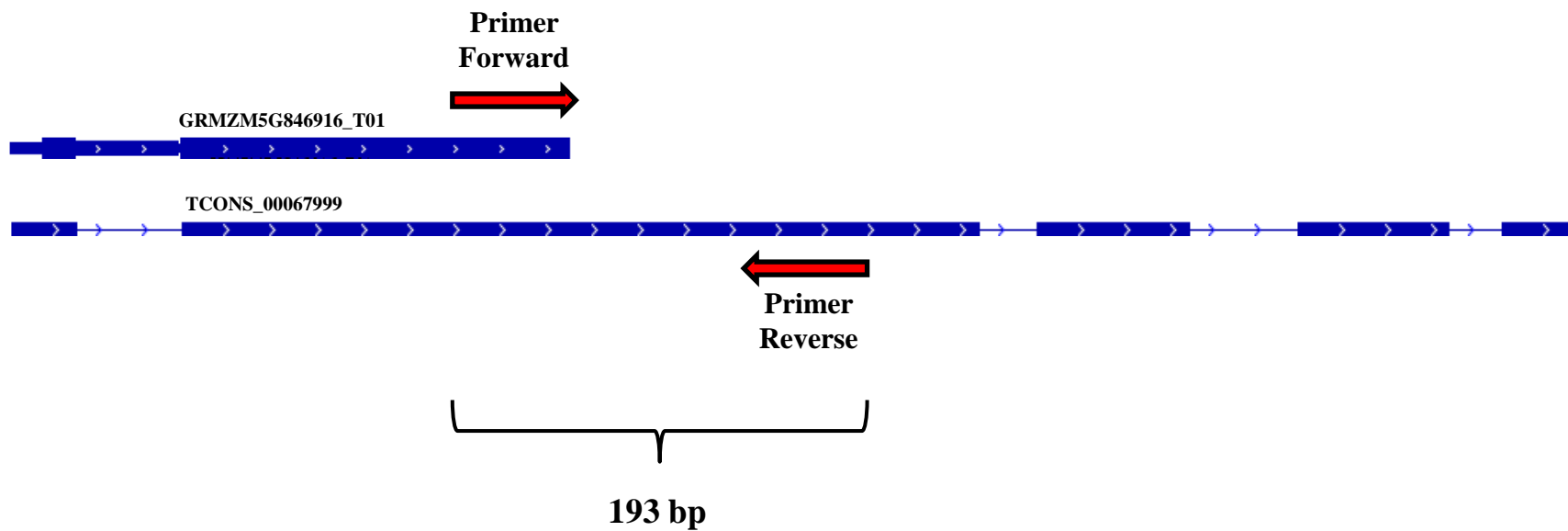

Supplement: Supplementary file 12 — Additional file 12: Figure S8: Semi-quantitative PCR expression profile of XLOC_029870 novel gene. The amplification product (193 bp) was reported for each biological replicate (numbered from 1 to 3) in CO354 genotype at 72 hpi. Primer forward was designed flanking the nucleotide region common to the gene GRMZ5G846916 and the predicted transcript TCONS_00067999, while reverse primer flanking the nucleotide region specific only to TCONS_00067999. L = 100 bp DNA ladder. (PDF 37 KB) [file 12864_2014_6392_MOESM12_ESM.pdf]

## Slide 1
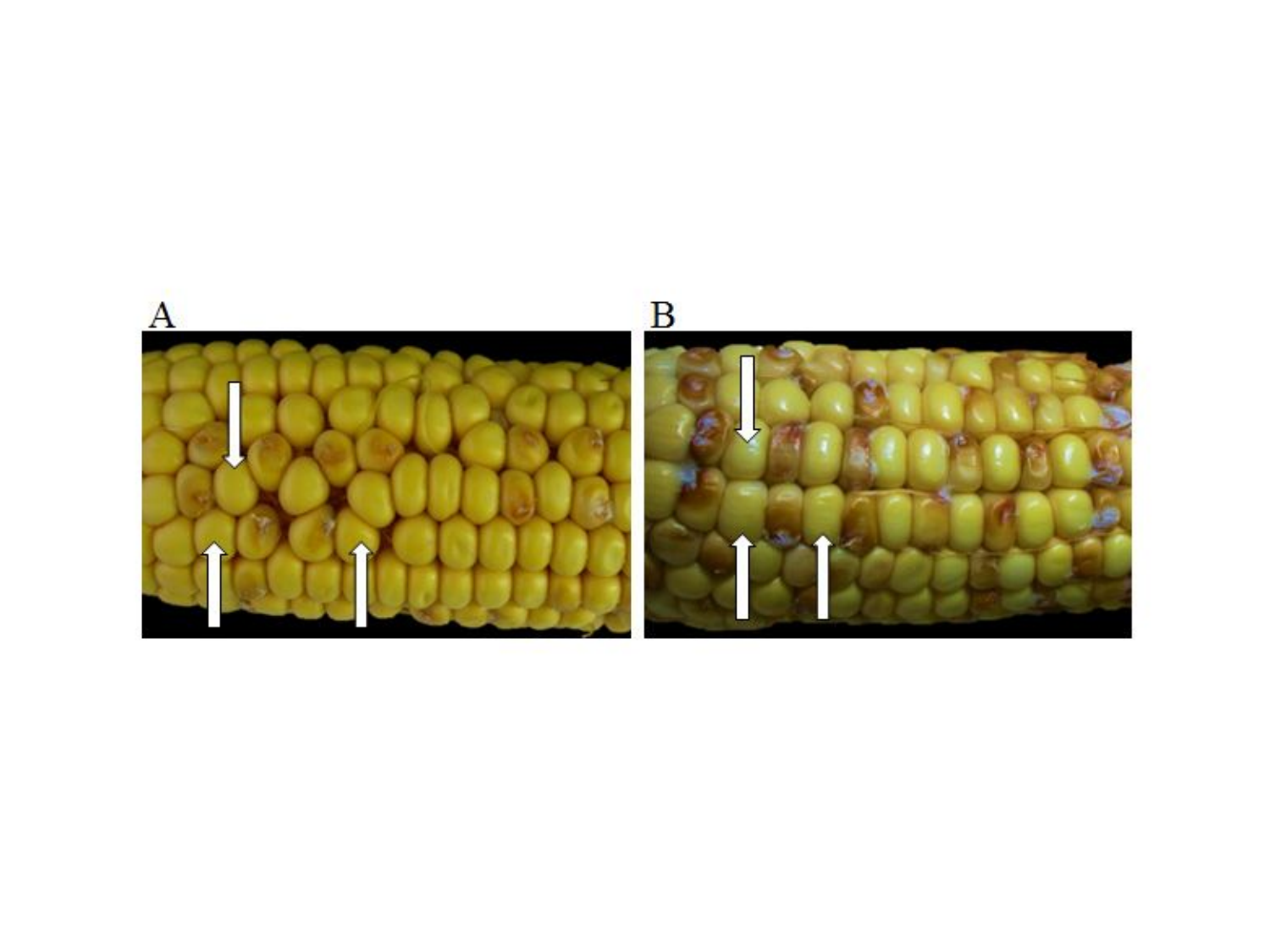

Supplement: Supplementary file 14 — Additional file 14: Figure S9: Ear of the susceptible maize line CO354 at 72 (A) and 96 (B) hpi with F. verticillioides. Arrows indicate examples of kernels sampled surrounding the inoculation points. (PPTX 131 KB) [file 12864_2014_6392_MOESM14_ESM.pptx]
