# Supplementary material for: Functional genomic analysis of constitutive and inducible defense responses to Fusarium verticillioides infection in maize genotypes with contrasting ear rot resistance
Source: BMC Genomics. 2014 Aug 25;15(1):710. doi: 10.1186/1471-2164-15-710 (PMC4153945; doi:10.1186/1471-2164-15-710)
Supplement: Supplementary file 7 — Additional file 7: Figure S3: Functional categories of differentially expressed genes modulated in CO441 and CO354 after F. verticillioides inoculation. DEGs in CO441 (A) and CO354 (B) at 72 hpi were annotated by Blast2GO analysis and classified in functional categories on the basis of literature evaluation. Induced genes are represented in light gray, while repressed ones are in black. The total percentage of modulated transcripts within each category is shown next to each bar. The complete list of genes is available in Additional file 6: Table S4. (PPTX 68 KB) [file 12864_2014_6392_MOESM7_ESM.pptx]

## Slide 1
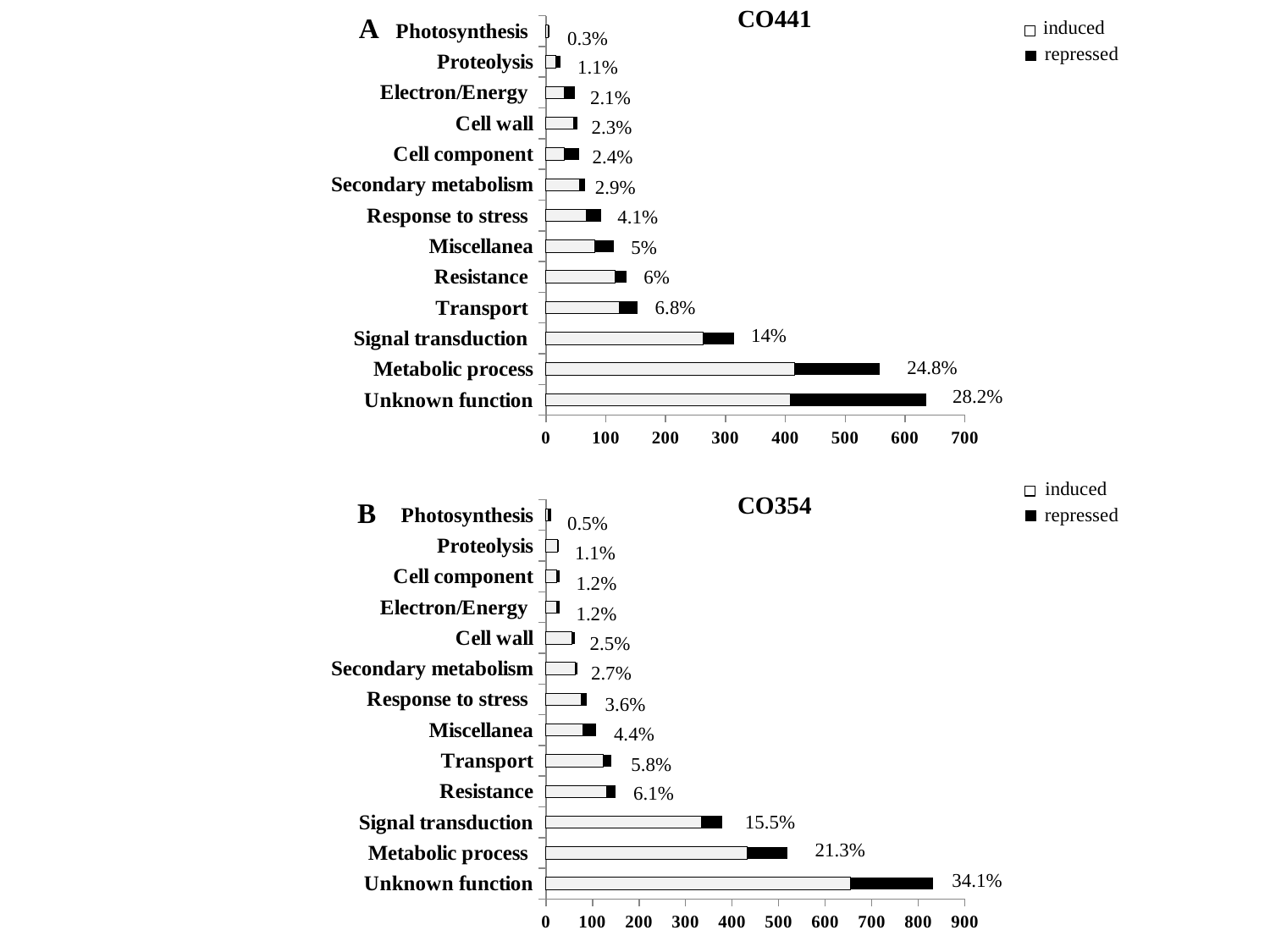

CO441
A
### Chart
| Category | Up | Down |
|---|---|---|
| Unknown function | 409.0 | 226.0 |
| Metabolic process | 415.0 | 142.0 |
| Signal transduction | 263.0 | 52.0 |
| Transport | 122.0 | 31.0 |
| Resistance | 115.0 | 19.0 |
| Miscellanea | 82.0 | 31.0 |
| Response to stress | 67.0 | 25.0 |
| Secondary metabolism | 56.0 | 10.0 |
| Cell component | 30.0 | 25.0 |
| Cell wall | 46.0 | 6.0 |
| Electron/Energy | 30.0 | 18.0 |
| Proteolysis | 17.0 | 7.0 |
| Photosynthesis | 4.0 | 2.0 |induced
repressed
0.3%
1.1%
2.1%
2.3%
2.4%
2.9%
4.1%
5%
6%
6.8%
14%
24.8%
28.2%
induced
repressed
CO354
B
### Chart
| Category | Up | Down |
|---|---|---|
| Unknown function | 654.0 | 178.0 |
| Metabolic process | 433.0 | 86.0 |
| Signal transduction | 335.0 | 44.0 |
| Resistance | 130.0 | 20.0 |
| Transport | 123.0 | 18.0 |
| Miscellanea | 79.0 | 28.0 |
| Response to stress | 76.0 | 11.0 |
| Secondary metabolism | 63.0 | 4.0 |
| Cell wall | 56.0 | 6.0 |
| Electron/Energy | 23.0 | 7.0 |
| Cell component | 23.0 | 6.0 |
| Proteolysis | 25.0 | 3.0 |
| Photosynthesis | 4.0 | 7.0 |0.5%
1.1%
1.2%
1.2%
2.5%
2.7%
3.6%
4.4%
5.8%
6.1%
15.5%
21.3%
34.1%
