# Supplementary material for: Functional genomic analysis of constitutive and inducible defense responses to Fusarium verticillioides infection in maize genotypes with contrasting ear rot resistance
Source: BMC Genomics. 2014 Aug 25;15(1):710. doi: 10.1186/1471-2164-15-710 (PMC4153945; doi:10.1186/1471-2164-15-710)
Supplement: Supplementary file 8 — Additional file 8: Figure S4: Distribution of common differentially expressed genes in CO441 and CO354 genotypes related to biotic stress processes, visualized by MapMan. Each square represents the FPKM expression value for one gene in control (heatmap on the left within each category) and inoculated (heatmap on the right within each category) resistant CO441 (A) and susceptible CO354 (B) genotypes. (PPTX 394 KB) [file 12864_2014_6392_MOESM8_ESM.pptx]

## Slide 1
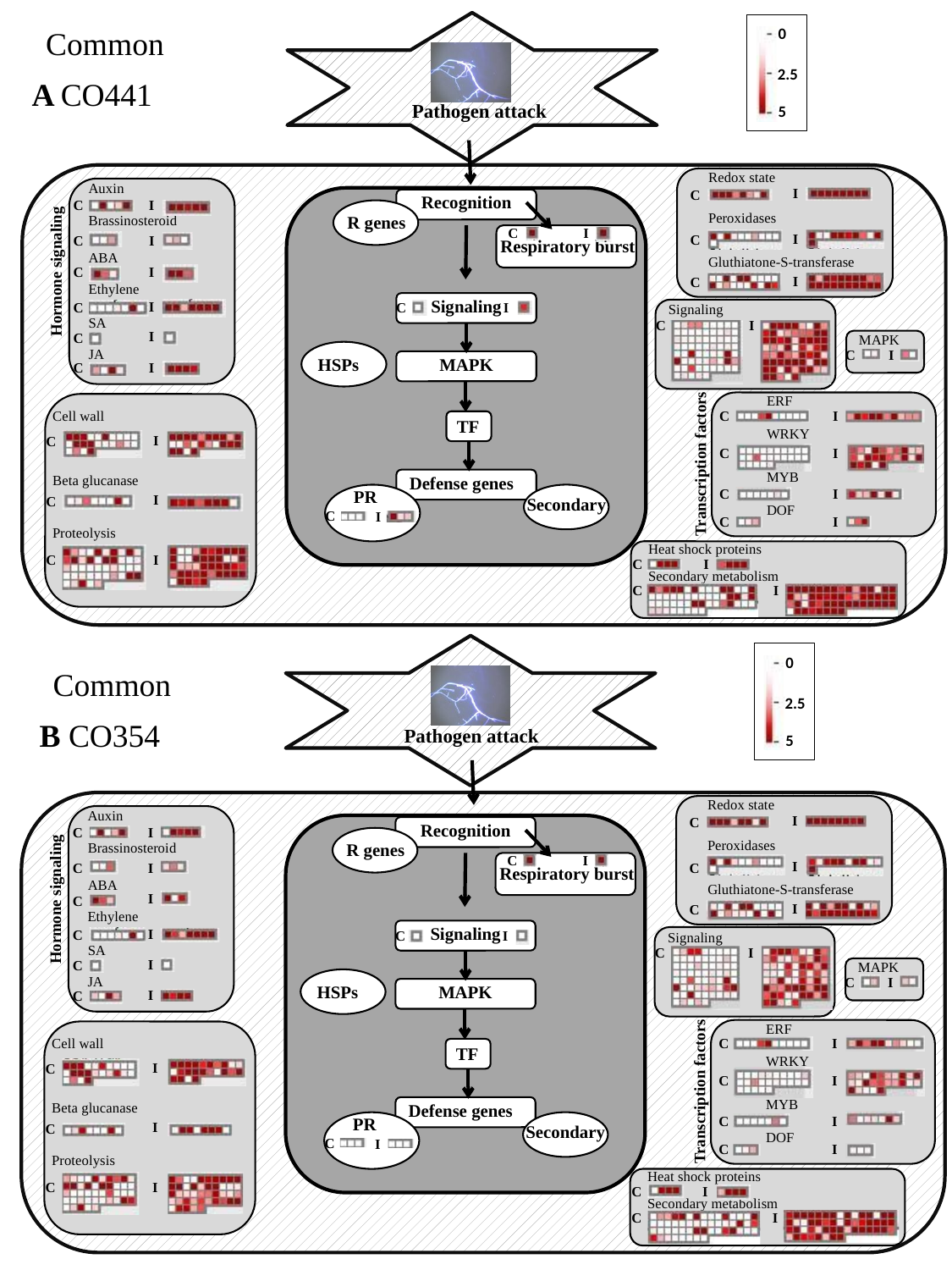

0
2.5
5
Common
A CO441
Pathogen attack
Redox state
Auxin
I
C
Recognition
C
I
Peroxidases
Brassinosteroid
R genes
C
I
I
C
C
I
Respiratory burst
ABA
Gluthiatone-S-transferase
Hormone signaling
C
I
I
C
Ethylene
Signaling
I
C
C
I
Signaling
SA
C
I
I
C
MAPK
JA
C
I
HSPs
MAPK
C
I
ERF
C
I
Cell wall
TF
WRKY
I
C
C
I
Transcription factors
MYB
Beta glucanase
Defense genes
C
I
PR
I
C
Secondary
DOF
C
I
C
I
Proteolysis
Heat shock proteins
C
I
C
I
Secondary metabolism
C
I
0
2.5
5
Common
B CO354
Pathogen attack
Redox state
Auxin
I
C
Recognition
C
I
Peroxidases
Brassinosteroid
R genes
C
I
I
C
C
I
Respiratory burst
ABA
Gluthiatone-S-transferase
Hormone signaling
I
C
I
C
Ethylene
Signaling
I
C
C
I
Signaling
SA
C
I
I
C
MAPK
JA
C
I
HSPs
MAPK
I
C
ERF
C
I
Cell wall
TF
WRKY
I
C
C
I
Transcription factors
MYB
Beta glucanase
Defense genes
C
I
PR
I
C
Secondary
DOF
C
I
C
I
Proteolysis
Heat shock proteins
C
I
C
I
Secondary metabolism
C
I
