# Supplementary material for: Functional genomic analysis of constitutive and inducible defense responses to Fusarium verticillioides infection in maize genotypes with contrasting ear rot resistance
Source: BMC Genomics. 2014 Aug 25;15(1):710. doi: 10.1186/1471-2164-15-710 (PMC4153945; doi:10.1186/1471-2164-15-710)
Supplement: Supplementary file 9 — Additional file 9: Figure S5: Comparison of RNA-Seq and real-time RT-PCR analyses for basal gene expression validation. Expression profiles of (A) heat shock protein 1 (GRMZM2G437100), (B) apoptosis inhibitor 5-like (GRMZM2G059039), (C) ethylene receptor (GRMZM2G420801), (D) germin-like protein 8-14-like (GRMZM2G343974), (E) protein peroxin-4-like (GRMZM2G461533), (F) phenylalanine ammonia-lyase (GRMZM2G118345), (G) terpene synthase (GRMZM2G028306), (H) calcium-dependent protein kinase (GRMZM2G047479), (I) pdr-like abc transporter (GRMZM2G445961). Histograms and errors bars represent normalized values relative to actin and standard deviations of three biological replicates, assessed by real-time RT-PCR in control kernels of the CO441 resistant line compared to the CO354 susceptible one, respectively. FPKM values are represented by dotted lines as assessed by RNA-Seq analysis. (PPTX 127 KB) [file 12864_2014_6392_MOESM9_ESM.pptx]

## Slide 1
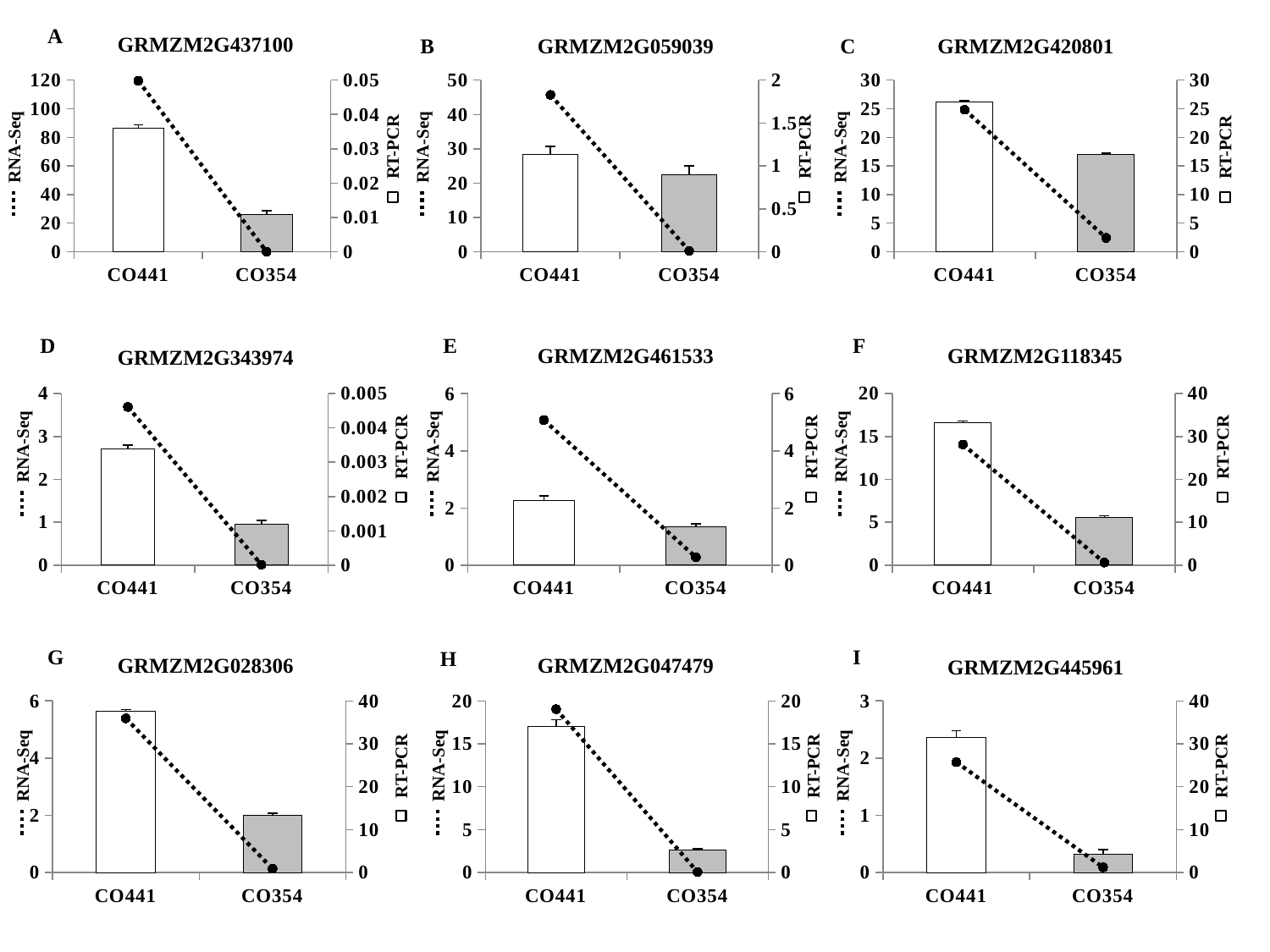

A
GRMZM2G437100
B
GRMZM2G059039
C
GRMZM2G420801
RT-PCR
RT-PCR
RT-PCR
RNA-Seq
### Chart
| Category | | |
|---|---|---|
| CO441 | 0.036 | 119.553 |
| CO354 | 0.0109 | 0.0453816 |RNA-Seq
### Chart
| Category | | |
|---|---|---|
| CO441 | 1.13 | 45.699 |
| CO354 | 0.9 | 0.260646 |RNA-Seq
### Chart
| Category | | |
|---|---|---|
| CO441 | 26.2 | 24.8243 |
| CO354 | 17.02 | 2.41938 |D
F
E
GRMZM2G461533
GRMZM2G118345
GRMZM2G343974
RT-PCR
RT-PCR
RT-PCR
RNA-Seq
RNA-Seq
RNA-Seq
### Chart
| Category | | |
|---|---|---|
| CO441 | 0.0034 | 3.68791 |
| CO354 | 0.0012 | 0.0107645 |
### Chart
| Category | | |
|---|---|---|
| CO441 | 2.25 | 5.0731 |
| CO354 | 1.34 | 0.276233 |
### Chart
| Category | | |
|---|---|---|
| CO441 | 33.12 | 14.0617 |
| CO354 | 11.06 | 0.300845 |G
I
H
GRMZM2G028306
GRMZM2G047479
GRMZM2G445961
RT-PCR
RT-PCR
RT-PCR
RNA-Seq
RNA-Seq
RNA-Seq
### Chart
| Category | | |
|---|---|---|
| CO441 | 37.53 | 5.39024 |
| CO354 | 13.36 | 0.131707 |
### Chart
| Category | | |
|---|---|---|
| CO441 | 31.55 | 1.93013 |
| CO354 | 4.3 | 0.0909148 |
### Chart
| Category | | |
|---|---|---|
| CO441 | 17.02 | 19.0317 |
| CO354 | 2.65 | 0.0601877 |
