# Supplementary material for: Functional genomic analysis of constitutive and inducible defense responses to Fusarium verticillioides infection in maize genotypes with contrasting ear rot resistance
Source: BMC Genomics. 2014 Aug 25;15(1):710. doi: 10.1186/1471-2164-15-710 (PMC4153945; doi:10.1186/1471-2164-15-710)
Supplement: Supplementary file 10 — Additional file 10: Figure S6: Comparison of RNA-Seq and real-time RT-PCR analyses after F. verticillioides inoculation. Expression profiles of (A) terpene synthase 5 (GRMZM2G127087), (B) thaumatin-like protein (GRMZM2G402631), (C) cysteine proteinase inhibitor (GRMZM2G012160), (D) pathogenesis-related maize seed protein (AC205274.3_FG001), (E) Chitinase 2-like (GRMZM2G358153), (F) glucosidase 5-like isoform (GRMZM2G055699), (G) cinnamoyl-reductase (GRMZM2G107076), (H) phenylalanine ammonia-lyase (GRMZM2G160541), (I) WRKY DNA-binding domain (GRMZM2G057116), (J) globulin-1 s allele precursor (GRMZM2G026703), (K) cinnamyl-alcohol dehydrogenase (GRMZM2G090980), (L) cell wall invertase (GRMZM2G119689), (M) phospholipase d (GRMZM2G140811), (N) beta-glucanase (GRMZM2G12503), (O) 4-diphosphocytidyl-2-c-methyl-d-erythritol kinase (GRMZM5G859195), (P) farnesyl pyrophosphate synthetase (GRMZM2G098569), (Q) lipoxygenase (GRMZM2G109056), (R) cysteine protease 1 (GRMZM2G073465), (S) lipoxygenase (GRMZM2G067225), (T) peroxisomal acyl-oxidase 1a (GRMZM5G864319), (U) o-methyltransferase zrp4 (GRMZM2G101735), (V) germin-like protein (GRMZM2G178817), (W) lipid binding protein (GRMZM2G155555), (X) 3-ketoacyl-thiolase peroxisomal-like (GRMZM2G169380). Dotted lines and histograms represent values expressed as fold change of transcript levels in the inoculated kernels with respect to the transcript levels in control maize samples for the CO441 and CO354 genotypes assessed by RNA-Seq and real-time RT-PCR analysis, respectively. The asterisk (*) means that the FC revealed by RNA-Seq method for that gene is not included among DEGs for one of the two genotypes. (PPTX 167 KB) [file 12864_2014_6392_MOESM10_ESM.pptx]

## Slide 1
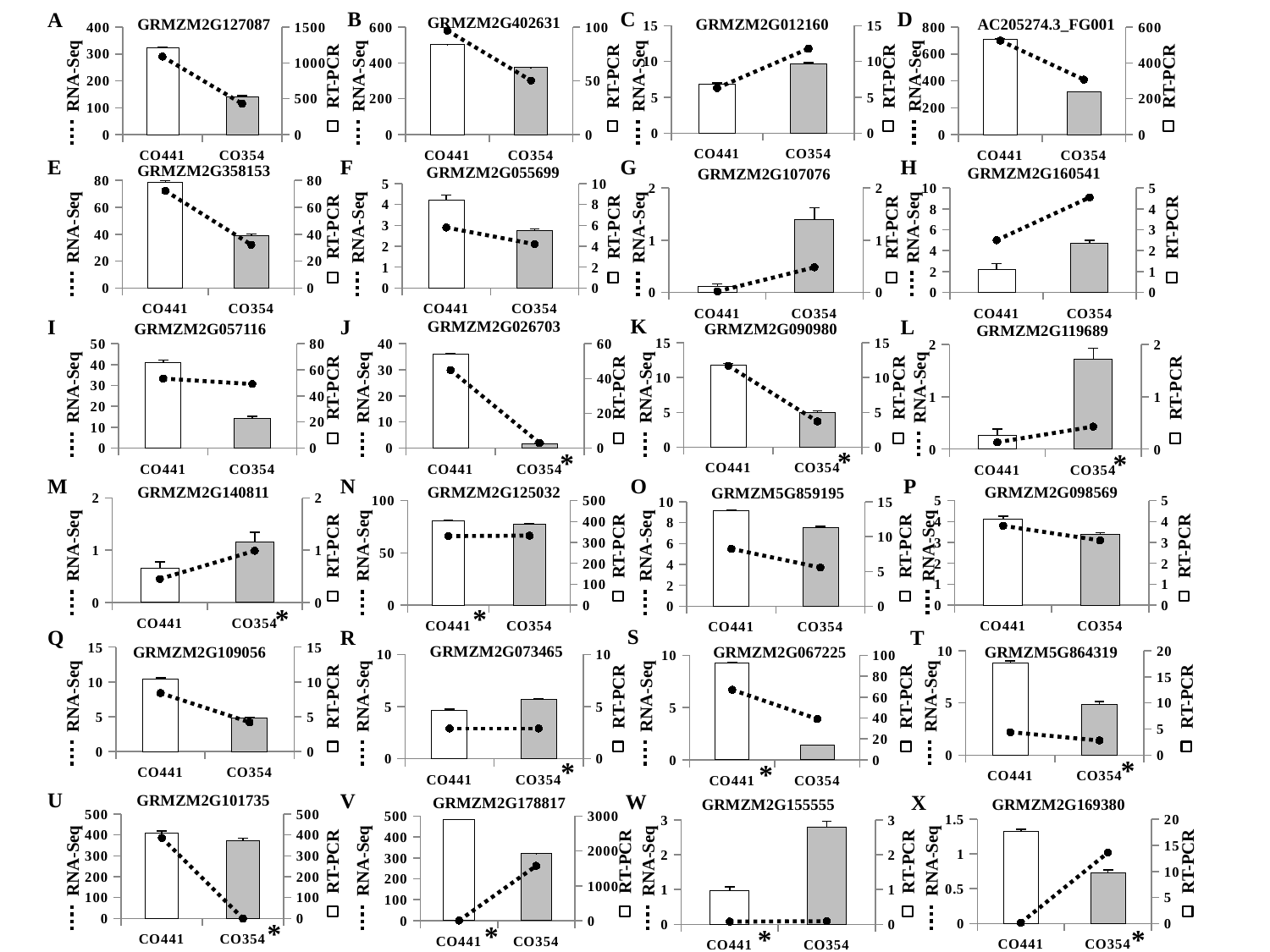

RT-PCR
RT-PCR
RT-PCR
RT-PCR
RNA-Seq
RNA-Seq
RNA-Seq
RNA-Seq
B
C
D
A
GRMZM2G402631
GRMZM2G127087
GRMZM2G012160
AC205274.3_FG001
### Chart
| Category | | |
|---|---|---|
| CO441 | 6.88 | 6.3 |
| CO354 | 9.71 | 11.8 |
### Chart
| Category | | |
|---|---|---|
| CO441 | 1212.81 | 290.2 |
| CO354 | 531.55 | 115.0 |
### Chart
| Category | | |
|---|---|---|
| CO441 | 83.69 | 580.6 |
| CO354 | 62.37 | 302.0 |
### Chart
| Category | | |
|---|---|---|
| CO441 | 532.85 | 699.2 |
| CO354 | 238.53 | 409.7 |RT-PCR
RT-PCR
RT-PCR
RT-PCR
RNA-Seq
RNA-Seq
RNA-Seq
RNA-Seq
E
F
G
H
GRMZM2G358153
GRMZM2G055699
GRMZM2G160541
GRMZM2G107076
### Chart
| Category | | |
|---|---|---|
| CO441 | 78.78 | 72.3 |
| CO354 | 39.02 | 32.1 |
### Chart
| Category | | |
|---|---|---|
| CO441 | 8.46 | 2.9 |
| CO354 | 5.54 | 2.1 |
### Chart
| Category | | |
|---|---|---|
| CO441 | 0.12 | 0.02 |
| CO354 | 1.4 | 0.48 |
### Chart
| Category | | |
|---|---|---|
| CO441 | 1.08 | 5.0 |
| CO354 | 2.37 | 9.1 |RT-PCR
RT-PCR
RT-PCR
RT-PCR
RNA-Seq
RNA-Seq
RNA-Seq
RNA-Seq
K
I
J
L
GRMZM2G026703
GRMZM2G057116
GRMZM2G090980
GRMZM2G119689
### Chart
| Category | | |
|---|---|---|
| CO441 | 11.84 | 11.7 |
| CO354 | 5.03 | 3.7 |
### Chart
| Category | | |
|---|---|---|
| CO441 | 53.83 | 29.9 |
| CO354 | 2.4 | 1.9 |
### Chart
| Category | | |
|---|---|---|
| CO441 | 65.59 | 33.3 |
| CO354 | 22.52 | 30.7 |
### Chart
| Category | | |
|---|---|---|
| CO441 | 0.26 | 0.13 |
| CO354 | 1.72 | 0.43 |*
*
*
RT-PCR
RT-PCR
RT-PCR
RT-PCR
RNA-Seq
RNA-Seq
RNA-Seq
RNA-Seq
M
N
O
P
GRMZM2G140811
GRMZM2G125032
GRMZM2G098569
GRMZM5G859195
### Chart
| Category | | |
|---|---|---|
| CO441 | 0.65 | 0.45 |
| CO354 | 1.15 | 0.99 |
### Chart
| Category | | |
|---|---|---|
| CO441 | 401.85 | 66.1 |
| CO354 | 387.14 | 66.6 |
### Chart
| Category | | |
|---|---|---|
| CO441 | 4.11 | 3.8 |
| CO354 | 3.37 | 3.1 |
### Chart
| Category | | |
|---|---|---|
| CO441 | 13.68 | 5.5 |
| CO354 | 11.35 | 3.7 |*
*
RT-PCR
RT-PCR
RT-PCR
RT-PCR
RNA-Seq
RNA-Seq
RNA-Seq
RNA-Seq
S
Q
T
R
GRMZM2G073465
GRMZM2G109056
GRMZM2G067225
GRMZM5G864319
### Chart
| Category | | |
|---|---|---|
| CO441 | 10.4 | 8.4 |
| CO354 | 4.76 | 4.2 |
### Chart
| Category | | |
|---|---|---|
| CO441 | 17.7 | 2.2 |
| CO354 | 9.73 | 1.4 |
### Chart
| Category | | |
|---|---|---|
| CO441 | 4.62 | 2.9 |
| CO354 | 5.68 | 2.9 |
### Chart
| Category | | |
|---|---|---|
| CO441 | 92.38 | 6.7 |
| CO354 | 13.75 | 3.9 |*
*
*
RT-PCR
RT-PCR
RT-PCR
RT-PCR
RNA-Seq
RNA-Seq
RNA-Seq
RNA-Seq
U
V
W
X
GRMZM2G101735
GRMZM2G178817
GRMZM2G155555
GRMZM2G169380
### Chart
| Category | | |
|---|---|---|
| CO441 | 408.86 | 386.1 |
| CO354 | 373.69 | 0.0 |
### Chart
| Category | | |
|---|---|---|
| CO441 | 2896.14 | 0.0 |
| CO354 | 1919.34 | 262.3 |
### Chart
| Category | | |
|---|---|---|
| CO441 | 17.7 | 0.012 |
| CO354 | 9.73 | 1.02 |
### Chart
| Category | | |
|---|---|---|
| CO441 | 0.98 | 0.08 |
| CO354 | 2.8 | 0.09 |*
*
*
*
