# Supplementary material for: Functional genomic analysis of constitutive and inducible defense responses to Fusarium verticillioides infection in maize genotypes with contrasting ear rot resistance
Source: BMC Genomics. 2014 Aug 25;15(1):710. doi: 10.1186/1471-2164-15-710 (PMC4153945; doi:10.1186/1471-2164-15-710)

## Slide 1
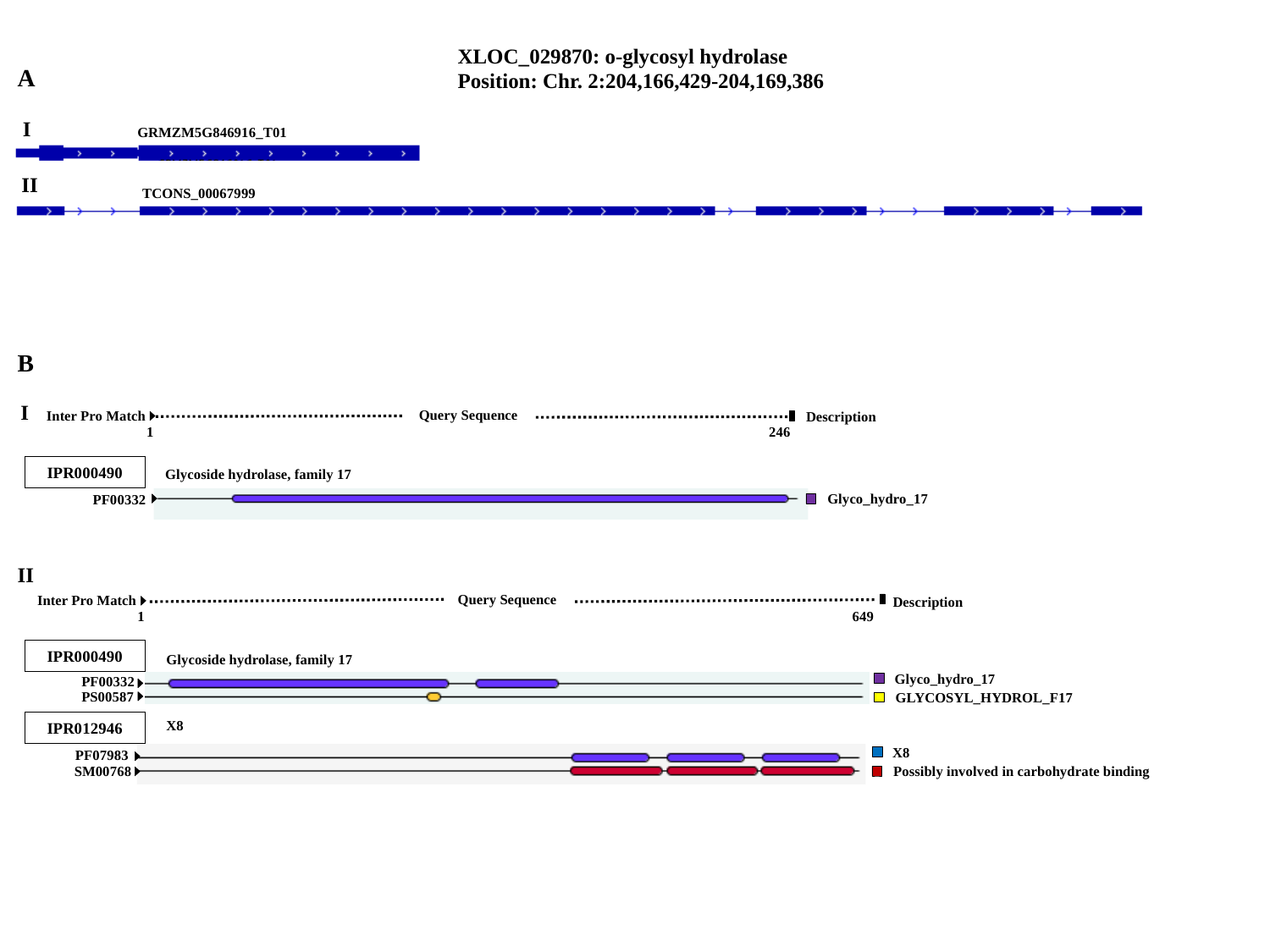

XLOC_029870: o-glycosyl hydrolase Position: Chr. 2:204,166,429-204,169,386
A
I
GRMZM5G846916_T01
II
TCONS_00067999
B
I
Query Sequence
Inter Pro Match
Description
1
246
IPR000490
Glycoside hydrolase, family 17
Glyco_hydro_17
PF00332
II
Query Sequence
Inter Pro Match
Description
1
649
IPR000490
Glycoside hydrolase, family 17
Glyco_hydro_17
PF00332
PS00587
GLYCOSYL_HYDROL_F17
X8
IPR012946
X8
PF07983
SM00768
Possibly involved in carbohydrate binding

## Slide 2
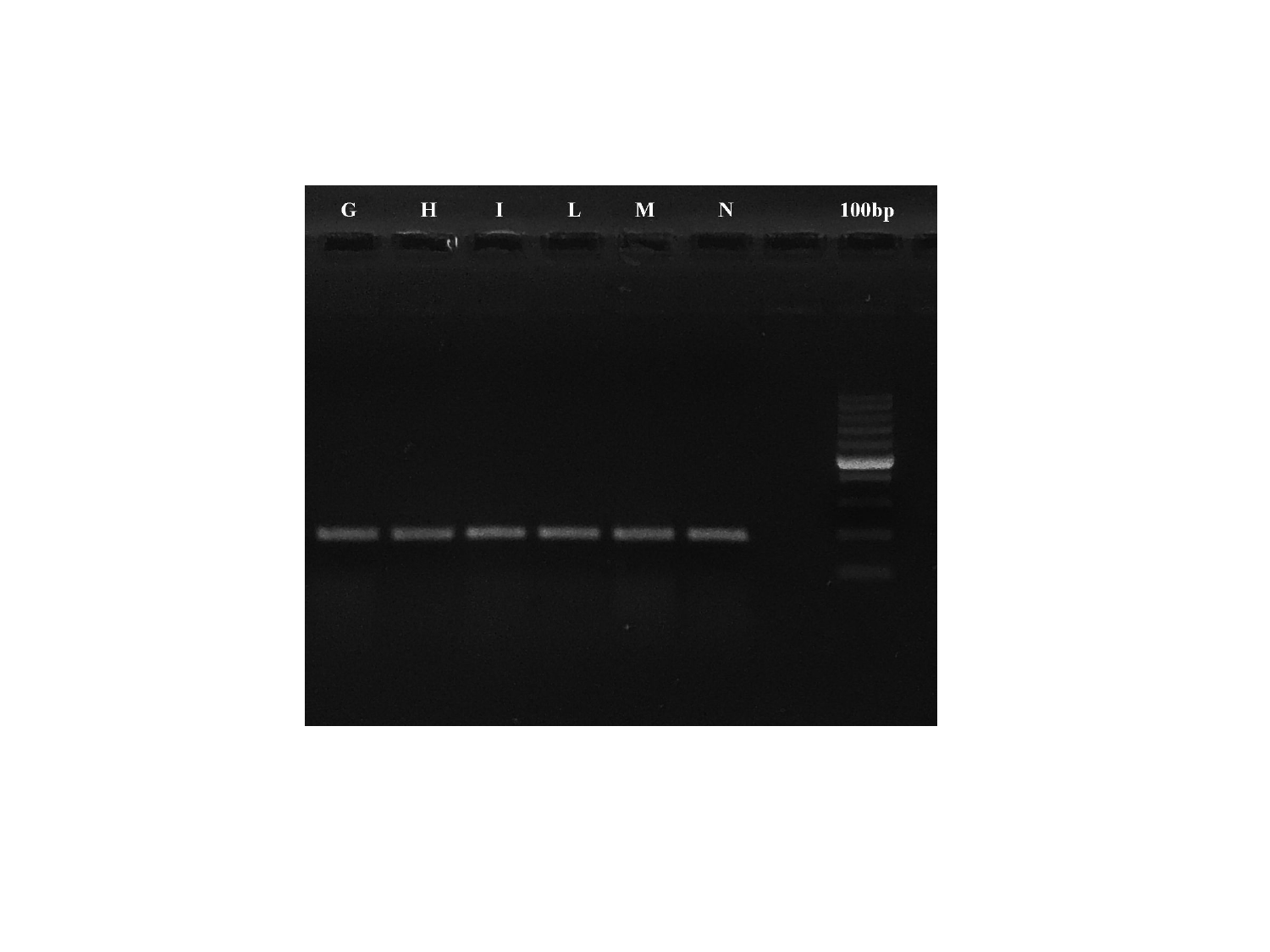

Supplement: Supplementary file 11 — Additional file 11: Figure S7: (A) Visualization of XLOC_029870 novel gene. Association of the GRMZ5G846916 annotated gene (I) with the predicted transcript TCONS_00067999 (II). (B) InterProScan protein structure. The 246-amino acids glycosyl hydrolase family 17 protein (ID: IPR000490) encoded by GRMZ5G846916 (I) and the 649-amino acids glycosyl hydrolase family 17 protein (IDs: IPR000490, IPR012946) encoded by XLOC_029870 are reported (II). The protein ID IPR012946 corresponds to the ×8 catalytic domain, possibly involved in carbohydrate binding and lacking in the annotated gene GRMZ5G846916. (PPTX 689 KB) [file 12864_2014_6392_MOESM11_ESM.pptx]
